# Supplementary material for: Population Genetics for Inferring Introduction Sources of the Oriental Fruit Fly, Bactrocera dorsalis: A Test for Quarantine Use in Korea
Source: Insects. 2021 Sep 22;12(10):851. doi: 10.3390/insects12100851 (PMC8541597; doi:10.3390/insects12100851)

# Population genetics for inferring introduction sources of the oriental fruit fly, *Bactrocera dorsalis*: A test for quarantine use in Korea

**Hyojoong Kim [1,*,†], Sohee Kim [1,2,†], Sangjin Kim [1], Yerim Lee [1,*], Heung-Sik Lee [2,*], Seong-Jin Lee [2], Deuksoo Choi [2], Jaeyong Jeon [2] and Jong-Ho Lee [2]**

[1] Animal Systematics Laboratory, Department of Biology, Kunsan National University, Gunsan, Jeonbuk 54150, Korea
[2] Animal & Plant Quarantine Agency, Gimcheon, Gyeongbuk 39660, Korea
[†] These authors contributed equally
[*] Correspondence: HK: hkim@kunsan.ac.kr; YL: yleeii@snu.ac.kr; H-S: lhsgo@korea.kr

**Supplementary material 1: Figures S1-S8**

**Figure S1.** The individual assignment result of 26 Korean samples from four quarantine-detected groups (KR 001-004) by STRUCTURE analysis ($K = 4$). According to Evanno et al. [90], the best delta $K$ was estimated to 4. The ordering number of individuals are consistent to that in Table S1.

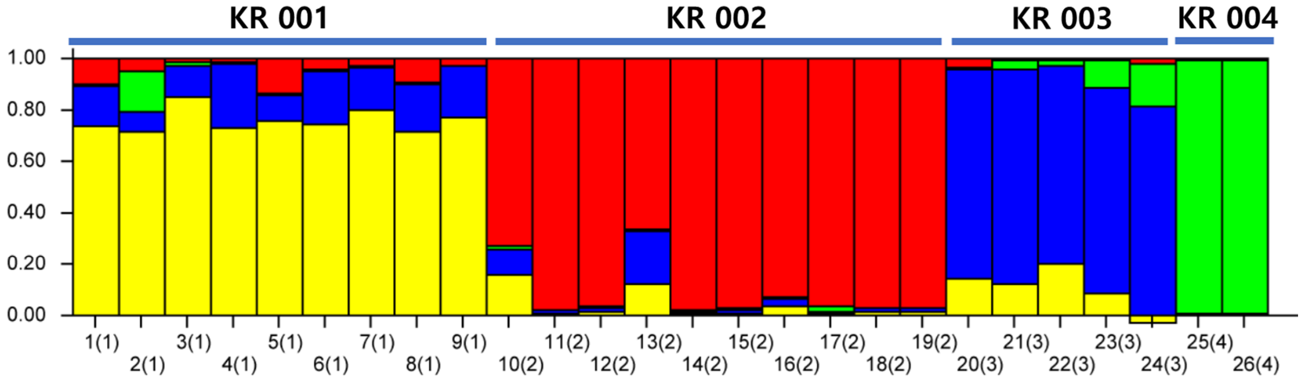

**Figure S2.** The simulated DIYABC historical scenarios by analysis (A). Six analysis scenarios to identify ancestral groups and to determine the separation order of each group. Each branch's color represents a specific group, red: Pop 1 (CH+VN), green: Pop 2 (TW+TH), blue: Pop 3 (PH+MY+IN). Information and results of scenarios 1-6 (see Table 3).

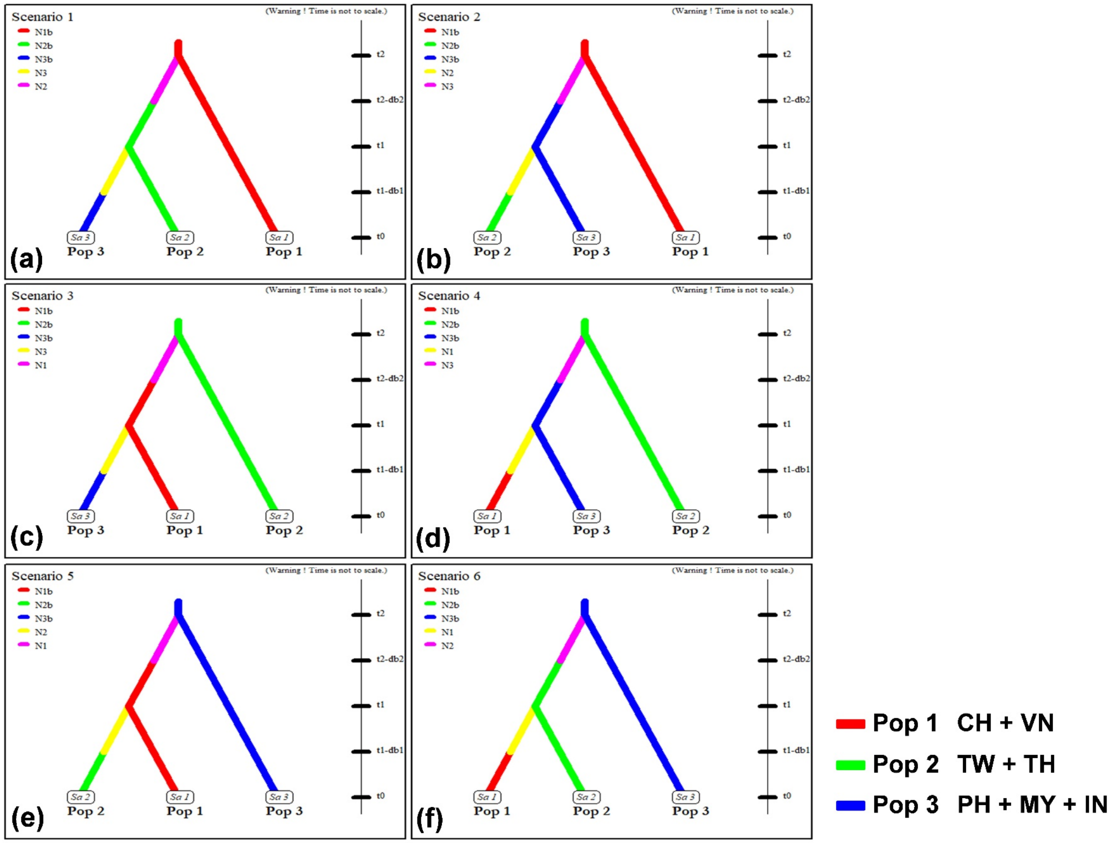

**Figure S3.** The simulated DIYABC historical scenarios by analysis B based on the former analysis A, Pop 2 (TW+TH) was fixed as an ancestral group, and we performed an analysis to determine from which country did the KR 001-003 originate. KR 001 and KR 002 were approached for the scenario 3 model (group KR 001 and 002 has a high probability of originating from Taiwan and Thailand). The KR 003 population has approached scenario 1 (KR 003 has a high probability of originating in China and Vietnam) (see Table 4).

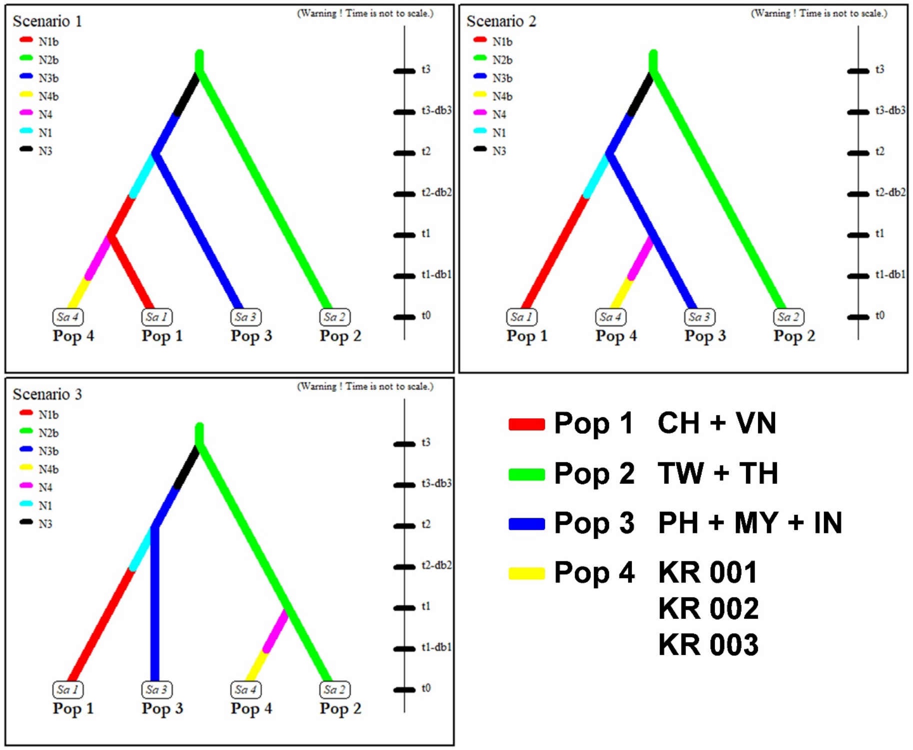

**Figure S4.** Allelic patterns in the 15 microsatellite loci in 40 populations of *Bactrocera dorsalis*. Na, number of different alleles; Na (Freq. ≥5%), number of alleles with a frequency greater than 5%; Ne, number of effective alleles; I, Shannon's Information Index; No. Private Alleles, number of alleles unique to a single population; No. LComm Alleles (≤25%), number of locally common alleles occurring in 25% or less in the populations; No. LComm Alleles (≤50%), number of locally common alleles occurring in 50% or less in the populations; He, expected heterozygosity; Vertical bars represent the standard error.

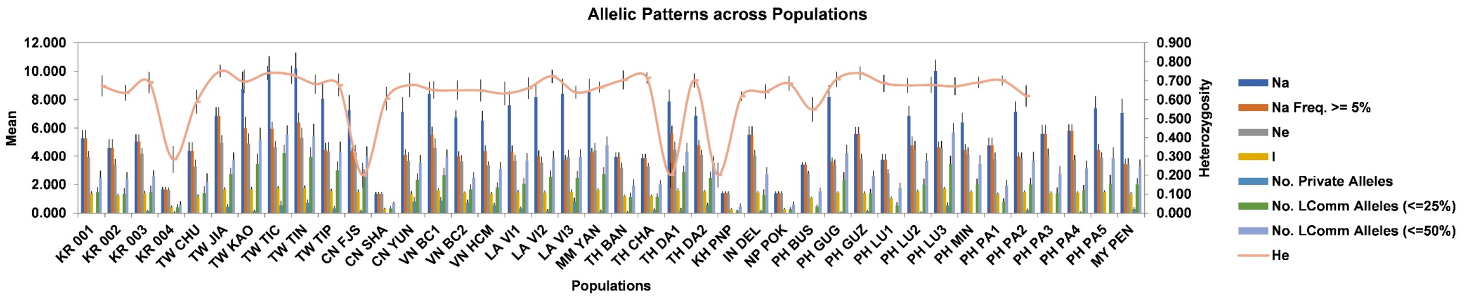

**Figure S5.** STRUCTURE Harvester results (approximate numbers). (a) Graph for the detection of the number of clusters (*K*) for observation of population group of *Bactrocera dorsalis*, realized by the STRUCTURE Harvester online software. Delta *K* values calculated by Evanno et al. [82] method detecting *K* = 2 groups. (b) Mean of probabilities lnP(*K*) and their standard deviation of the posterior probability.

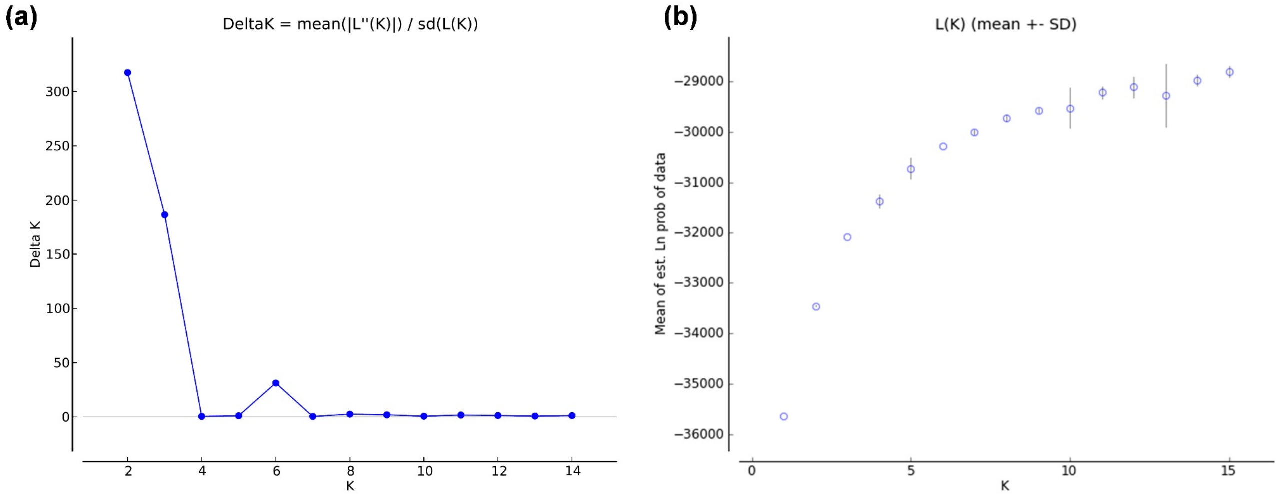

**Figure S6.** Bayesian clustering by STRUCTURE. Individual assignment plots for $K = 2, 3, 4, 5, 6$. Mean log probability of the data for different $K$ values (1–15), and plot of $\Delta K$ statistic of Evanno et al. [83] showing best $K = 2$. (a) In the result of $K = 2$, KR 001,002, TW, and TH showed the same clustering, and the other groups were separated. (b) The result of $K = 3$ was the same as that of PCoA. KR 001, 002 have the same clustering as TW+TH, whereas KR 003 has the same clustering as CH+VN (BC1, 2)+LA+MY.

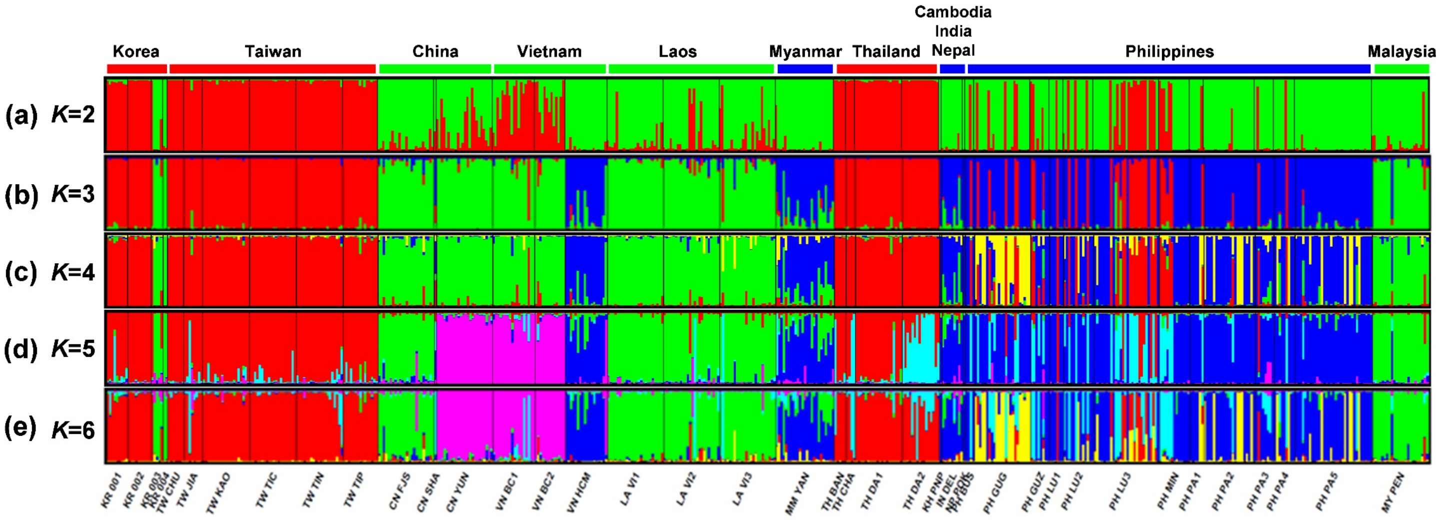

**Figure. S7.** Results of the DIYABC analysis 'A'. Direct estimate; left, Logistic regression; right. Scenarios 1 to 6 are color labeled (Table 3, Figure S2).

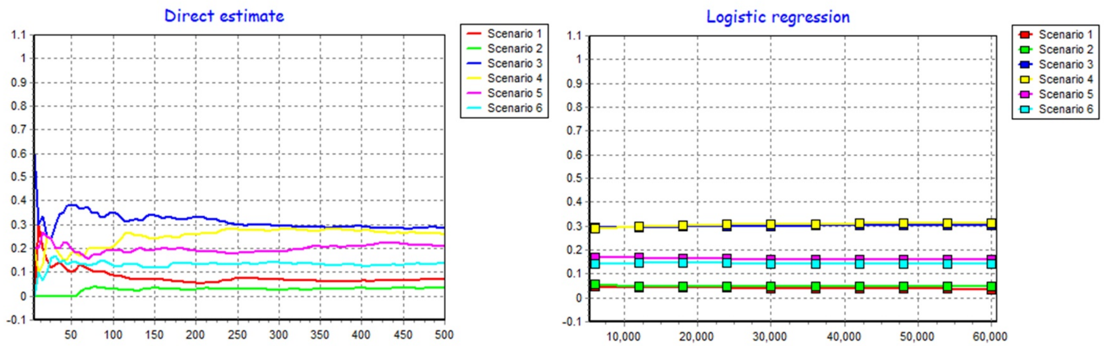

**Figure S8.** Results of the DIYABC analysis 'B'. The B-1 is the analysis result for the identification of the origin of KR 001. The B-2 is the analysis result for the identification of the origin of KR 002. The B-3 is the analysis result for the identification of the origin of KR 003. Each color reflects each evolutionary scenario in the legend on the right. The red legend on each graph (scenario 1) is a scenario where CN+VN is the origin, the green legend (scenario 2) means TW+TH is the origin, and the blue legend (scenario 3) means PH+MY+IN is the origin (Table 4, Figure S3). KR 001 and 002 have the highest logistic regression of scenarios originated from TW+TH, KR 003 has the highest logistic regression of the scenario originated from CN+VN.

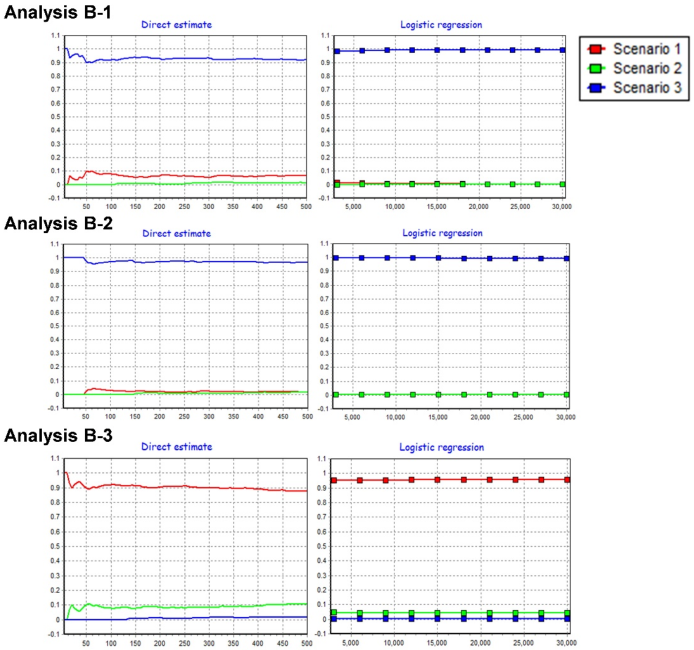

Supplement: Supplementary file 1 [file insects-12-00851-s001.zip › _Supplementary material 1 Figures S1-S8_REV2.pdf]
