# Supplementary material for: Population Genetics for Inferring Introduction Sources of the Oriental Fruit Fly, Bactrocera dorsalis: A Test for Quarantine Use in Korea
Source: Insects. 2021 Sep 22;12(10):851. doi: 10.3390/insects12100851 (PMC8541597; doi:10.3390/insects12100851)
Supplement: Supplementary file 1 [file insects-12-00851-s001.zip › _Supplementary material 2 Tables S1-S7_REV1.pdf]

Population genetics for inferring introduction sources of the oriental fruit fly, *Bactrocera dorsalis*: A test for quarantine use in Korea

Hyojoong Kim [1,*,†], Sohee Kim [1,2,†], Sangjin Kim [1], Yerim Lee [1,*], Heung-Sik Lee [2,*], Seong-Jin Lee [2], Deuksoo Choi [2], Jaeyong Jeon [2] and Jong-Ho Lee [2]

[1] Animal Systematics Laboratory, Department of Biology, Kunsan National University, Gunsan, Jeonbuk 54150, Korea
[2] Animal & Plant Quarantine Agency, Gimcheon, Gyeongbuk 39660, Korea
† These authors contributed equally
* Correspondence: HK: hkim@kunsan.ac.kr; YL: yleeii@snu.ac.kr; H-S: lhsgo@korea.kr

Supplementary material 2: Tables S1-S7

Table S1. Collection data for oriental fruit fly (*Bactrocera dorsalis*) analyzed in this study. † Abbreviations for collectors: APQA = Animal and plant quarantine agency of Korea; JJ = Jaeyong Jeon; HN = Hyeban Namgung; HK = Hyojoong Kim; DC = Deuksu Choi; SL = Seong-Jin Lee; JL = Jong-Ho Lee.

Pop. ID	Country	Collection site	No.	Latitude, longitude	Date (DD/MM/YYYY)	Collected by†
		Quarantine-detected group 1	9			
		(Siheung, Gyeonggi)	(3)		02/09/2016	
KR 001	Korea	(Ansan, Gyeonggi)	(1)	N/A	07/06/2016	APQA
		(Gimhae, Gyeongbuk)	(3)		24/08/2016	
		(Incheon, Gyeonggi)	(2)		11/09/2015, 06/10/2015	
KR 002	Korea	Quarantine-detected group 2	10	N/A		APQA
		(Jeju, Jeju)	(10)		26/09/2015~16/10/2015	
		Quarantine-detected group 3	5			
KR 003	Korea	(Seogwipo, Jeju)	(4)	N/A	2015~2017	APQA
		(Uijeongbu, Gyeonggi)	(1)		18/07/2017	
KR 004	Korea	Quarantine-detected group 4	2	N/A		APQA
		(Uijeongbu, Gyeonggi)			03/08/2017	
TW CHU	Taiwan	Chung Hsing university, Taichung	7	24°07'23.5"N 120°40'32.9"E	18/11/2016	APQA
TW JIA	Taiwan	City of Jiayi (Chiayi)	8	23°26′29.2″N 120°30′29.4″E	10/11/2016	APQA
TW KAO	Taiwan	Kaohsiung	20	22°44′06.5″N 120°22′23.6″E	09/11/2016	APQA
TW TIC	Taiwan	Taichung City	20	24°08′08.9″N 120°41′08.9″E	10/11/2016	APQA
TW TIN	Taiwan	Tainan, Dōngshān Qū	20	23°17′13.8″N 120°23′57.6″E	09/11/2016	APQA
TW TIP	Taiwan	Taipei City	15	25°01'47.0"N 121°32'08.7"E	11/11/2016	APQA
CN FJS	China	Fújiàn Shěng	24	24°53'11.0"N 118°39'17.6"E	28/08/2016	JJ
CN SHA	China	Shànghǎi,	1	31°13'50.5"N 121°28'22.2"E	28/08/2016	JJ
CN YUN	China	Yúnnán Shěng	24	23°34'21.6"N 104°19'36.9"E	08/08/2017	JJ
VN BC1	Vietnam	Bờ hồ, Cúc Phương	18	20°15'30.7"N 105°42'26.6"E	09/05/2017	HN
VN BC2	Vietnam	Bờ hồ, Cúc Phương	13	20°15'30.7"N 105°42'26.6"E	11/05/2017	HN
VN HCM	Vietnam	Hồ Chí Minh	18	10°46'37.3"N 106°41'00.5"E	10/12/2015	HK
LA VI1	Laos	Vientiane	24	17°58'32.1"N 102°37'53.2"E	14/12/2016	APQA
LA VI2	Laos	Vientiane	24	17°58'32.1"N 102°37'53.2"E	04/01/2017	APQA
LA VI3	Laos	Vientiane	24	17°58'32.1"N 102°37'53.2"E	01/02/2017	APQA
MM YAN	Myanmar	Yangan	25	16°49'59.7"N 96°07'12.5"E	25/01/2016	SL
TH BAN	Thailand	Maruay Garden Hotel	5	13°50'42.8"N 100°34'47.8"E	28/11/2016	APQA
TH CHA	Thailand	Plant Pest Surveillance Section	4	12°37'06.4"N 102°06'58.0"E	28/11/2016	APQA
TH DA1	Thailand	Department of Agriculture	20	13°50'53.9"N 100°34'26.1"E	28/11/2016	APQA
TH DA2	Thailand	Department of Agriculture	16	13°50'53.9"N 100°34'26.1"E	29/11/2016	APQA
KH PNP	Cambodia	Phnom penh	1	11°33'37.4"N 104°55'18.5"E	11/09/2016	SL
IN DEL	India	Delhi	9	28°35'49.7"N 77°11'47.9"E	09/11/2016	APQA
NP POK	Nepal	Pokhara	1	28°14'05.2"N 83°59'44.9"E	20/09/2016	DC
PH BUS	Philippines	Busuanga	4	12°09'28.6"N 119°55'08.4"E	19/12/2015	SL
PH GUG	Philippines	Guimarasas	24	10°36'10.1"N 122°35'19.1"E	18/12/2015	SL
PH GUZ	Philippines	Guimaras Zempkamps	8	10°36'11.5"N 122°35'20.0"E	19/12/2015	SL
PH LU1	Philippines	Manila PEQS	6	14°35'07.1"N 120°58'34.5"E	16/12/2015	SL
PH LU2	Philippines	Manila Tropicana Suites	13	14°34'28.6"N 120°59'12.2"E	17/12/2015	SL
PH LU3	Philippines	Manila	28	14°35'07.1"N 120°58'34.5"E	20/12/2015	SL
PH MIN	Philippines	Zamboanga	13	6°55'45.1"N 122°06'16.6"E	21/12/2015	SL
PH PA1	Philippines	ILOILO NPQS Station	6	10°43'08.2"N 122°34'49.5"E	17/12/2015	SL
PH PA2	Philippines	ILOILO NPQS Station	22	10°43'08.2"N 122°34'49.5"E	19/12/2015	SL
PH PA3	Philippines	ILOILO LEON Province	8	10°46'51.4"N 122°23'19.5"E	19/12/2015	SL
PH PA4	Philippines	ILOILO PAVIA	9	10°46'05.0"N 122°32'08.8"E	19/12/2015	SL
PH PA5	Philippines	ILOILO	33	10°42'16.3"N 122°33'03.4"E	20/12/2015	SL
MY PEN	Malaysia	Pinang	24	5°21'37.8"N 100°18'19.1"E	07/07/2017	JL

Table S2. Microsatellite loci and primer sequences and multiplex PCR primer set used in this study. Set; unit of multiplex PCR and capillary electrophoresis injection.

Set	No.	Locus	Repeat motif	Primer sequence 5'-3'	Size range (in bp)	Tm℃	NA	GenBank Accession code	References
(A)	1	Bp200_FAM	$(CA)_5$	CGTTACGTTGGTTGCACGTAG	108–110	61.3	4	AY847282	Shearman *et al*. 2006
		Bp200_R		CAACACGGCCACACTTCATTG		61.3			
	2	Bi4_VIC	$(CT)_6GCT$	GCACTCGCATGCTTGTAGTC	123-126	60.5	4	EU868615	Khamis *et al*. 2008
		Bi4_R		CCGGTTTTGTGCGAAAAG		53.9			
(B)	3	Bt5.8a_NED	$(TG)_9$	CACGACGTATGATTTCAATTGC	133	58.4	4		Wang *et al*. 2003
		Bt5.8a_R		GCTTACTGCAATTCCCACTTC		59.4			
	4	Bt4_FAM	$(GT)_8$	AGTGGGTGTTGCTCACTTAC	120-126	58.4	4		Kinnear *et al*. 1998
		Bt4_R		AATGGCTAACGTTGCCGCGA		60.5			
	5	Bi8_VIC	$(AC)_5AT(AC)_2$	ACAAGTGCAGCAAAGACACG	118-138	58.4	7	EU868619	Khamis *et al*. 2008
		Bi8_R		ATCACATCATGAGGCGTTCA		56.4			
	6	Bt6.8a_NED	$(CAT)_5$	AGCAACACTATAGGCTGGTC	147	58.4	3		Wang *et al*. 2003
		Bt6.8a_R		GTAACGCGTTACTGTCATTG		56.4			
	7	Boms3a_PET	$(TA)_2TG(TA)CA(TA)_3$ $A_2(TG)_5CA(TG)(TC)_2(TA)_2(TG)_7$	CAGTCGCCCTTTAATTTGC	176	55.2	4	EU489747	Augustinos *et al*. 2008
		Boms3a_R		GGGTCCTTTTGTTCTCAGG		57.3			
(C)	8	Bi7_FAM	$(CA)_5A(CA)_2$	CTCGCTCTTCATTCAATCCA	107-118	56.4	5	EU868618	Khamis *et al*. 2008
		Bi7_R		CGACACGTTAAGTGGCAAAA		56.4			
	9	Bt9.1a_VIC	$(AC)_{11}$	ACAAATGGCGTGGCATGTC	142	57.3	5		Wang *et al*. 2003
		Bt9.1a_R		TGTGCGCTAACAATCTAACGC		59.4			
	10	Bt7.2b_NED	$(CA)_6CG(CA)_3$	GCGATCCAATTTCCGAATAAC	147	57.4	3		Wang *et al*. 2003
		Bt7.2b_R		CAATTGCCAGCAATAAGTGG		56.4			
	11	Bi11_PET	$(TC)_3T(TC)_3$	TGGGTTCACCGTCCTTTAAT	140-147	56.4	3	EU868622	Khamis *et al*. 2008
		Bi11_R		GCCCATAGACATCCAGGGTA		60.5			
(D)	12	Bp181_FAM	$(AC)_8$	AAAGGTGCATGCCTTCGTGTAG	112–126	62.1	4	AY847280	Shearman *et al*. 2006
		Bp181_R		TGGCTTCATCGATGAATCTGCG		62.1			
	13	Bt4.6a_VIC	$(GT)_8$	GCATGCATGTGACAAGGAGA	137	58.4	6		Wang *et al*. 2003
		Bt4.6a_R		CCATGTACAGCCGAGGTAAATG		62.1			
	14	Boms60_NED	$CACA_2(CA3)_2$/ $A_{13}CA_6TA_4N_4A_3CA_2TA_2CA_5$	TGGACGCGCACATATCAG	170	56.1	6	DQ078251	Augustinos *et al*. 2008
		Boms60_R		ACGACGTTTAGCGGAAATGAG		59.4			
	15	Bt5_PET	$(CA)_9$	GATCACCCATCAATCGTTATA	181-187	55.5	4		Kinnear *et al*. 1998
		Bt5_R		TAAGCAGATGCCTATATGTTG		55.5			

Table S3. 465 *COI* haplotypes split into 40 populations. The number of samples belonging to each haplotype is displayed in the columns under population ID code (see Table S1).

Country	Korea				Taiwan						China			Vietnam			Laos			Myanmar
	KR 001	KR 002	KR 003	KR 004	TW CHU	TW JIA	TW KAO	TW TIC	TW TIN	TW TIP	CN FJS	CN SHA	CN YUN	VN BC1	VN BC2	VN HCM	LA VI1	LA VI2	LA VI3	MM YAN
H1	8	10	4			6		16	4	13	22					14	21	20	22	19
H2	1							1	1								1			
H3			1							1										1
H4				2																
H5						1														
H6						1														
H7								1												
H8								1												
H9									1											
H10									1											
H11										1										
H12										1										
H13											2									
H14											1									
H15															3					
H16																	1			
H17																	1			
H18																		1		
H19																		1		
H20																			1	
H21																				1
H22																				1
H23																				1
H24																				1
H25																				
H26																				
H27																				
H28																				
H29																				
H30																				
H31																				
H32																				
H33																				
H34																				
H35																				
H36																				
H37																				
H38																				
H39																				
H40																				
H41																				
H42																				
H43																				
H44																				
H45																				
H46																				
H47																				
H48																				
H49																				
H50																				
Number of Haplotype	2	1	2	1	0	3	0	4	4	4	3	0	0	0	1	1	4	3	2	6

Table S3. Continued.

Country	Thailand				Cambodia	India	Nepal	Philippines												Malaysia
	TH BAN	TH CHA	TH DA1	TH DA2	KH PNP	IN DEL	NP POK	PH BUS	PH GUG	PH GUZ	PH LU1	PH LU2	PH LU3	PH MIN	PH PA1	PH PA2	PH PA3	PH PA4	PH PA5	MY PEN
H1	3	3	10	16	1	5	1	3		2	6	7	11	6	4	13	4	4	23	11
H2																				1
H3			1																	
H4																				
H5																				
H6																				1
H7																				1
H8																				
H9																				
H10																				
H11																				
H12																				
H13																				
H14																				
H15																				
H16																				
H17		1	1																	
H18									1								1		1	
H19										1						2		1	1	
H20																				
H21																				
H22																				
H23						1													1	
H24																				2
H25	1																			
H26	1																			
H27			8																	
H28						1														1
H29						1														
H30						1														
H31								1	5	2		3	9	4		1	2	2	1	
H32									18			2	4		2	5		2	4	
H33									1											
H34										1										
H35										1										
H36										1										
H37													1							
H38													1							
H39													1							
H40													1							
H41														1						
H42														2			1			
H43																1				
H44																			1	
H45																			1	
H46																				2
H47																				2
H48																				1
H49																				1
H50																				1
Number of Haplotype	3	2	4	1	1	5	1	2	4	6	1	3	7	4	2	5	4	4	8	11

Table S4. Pairwise F_{ST} divergence between 39 different geographical populations of the oriental fruit fly (*B. dorsalis*). Values are significantly different from zero at $P < 0.001$ unless indicated ‘ns’. CN SHA, KH PNP, NP POK are omitted from the table because they are 'NA: non-available' due to lack of individuals.

	KR 001	KR 002	KR 003	KR 004	TW CHU	TW JIA	TW KAO	TW TIC	TW TIN	TW TIP	CN FJS	CN YUN	VN BC1	VN BC2	VN HCM	LA VI1	LA VI2	LA VI3	MM YAN
KR 001	0.000																		
KR 002	0.071	0.000																	
KR 003	0.044	0.105	0.000																
KR 004	0.201	0.271	0.124	0.000															
TW CHU	0.063	0.105	0.074	0.250	0.000														
TW JIA	0.013	0.077	0.014	0.143	0.037	0.000													
TW KAO	0.022	0.068	0.055	0.179	0.025	0.024	0.000												
TW TIC	0.041	0.076	0.051	0.200	0.050	0.021	0.017	0.000											
TW TIN	0.026	0.065	0.032	0.163	0.049	0.012	0.018	0.018	0.000										
TW TIP	0.042	0.088	0.067	0.224	0.056	0.034	0.011	0.004	0.030	0.000									
CN FJS	0.063	0.127	0.016	0.113	0.107	0.073	0.083	0.097	0.080	0.102	0.000								
CN YUN	0.130	0.182	0.086	0.210	0.179	0.133	0.136	0.153	0.126	0.158	0.080	0.000							
VN BC1	0.075	0.124	0.069	0.108	0.119	0.064	0.077	0.097	0.081	0.095	0.067	0.102	0.000						
VN BC2	0.093	0.157	0.076	0.133	0.142	0.083	0.103	0.117	0.104	0.115	0.085	0.110	0.013	0.000					
VN HCM	0.149	0.215	0.150	0.187	0.214	0.154	0.169	0.173	0.172	0.182	0.135	0.211	0.129	0.135	0.000				
LA VI1	0.054	0.128	0.038	0.156	0.105	0.059	0.087	0.082	0.082	0.081	0.052	0.106	0.100	0.117	0.159	0.000			
LA VI2	0.051	0.151	0.047	0.196	0.116	0.074	0.099	0.105	0.094	0.097	0.066	0.125	0.114	0.121	0.141	0.008	0.000		
LA VI3	0.044	0.121	0.039	0.156	0.099	0.058	0.081	0.088	0.078	0.083	0.057	0.123	0.106	0.120	0.140	0.007	0.006	0.000	
MM YAN	0.090	0.154	0.078	0.192	0.146	0.104	0.129	0.128	0.115	0.143	0.093	0.149	0.121	0.132	0.059	0.112	0.102	0.106	0.000
TH BAN	0.028	0.100	0.086	0.258	0.104	0.037	0.058	0.052	0.054	0.054	0.132	0.211	0.099	0.125	0.167	0.118	0.120	0.107	0.127
TH CHA	0.056	0.121	0.100	0.296	0.143	0.059	0.094	0.055	0.083	0.068	0.137	0.214	0.121	0.132	0.180	0.113	0.124	0.105	0.122
TH DA1	0.019	0.068	0.061	0.191	0.074	0.039	0.036	0.021	0.027	0.023	0.092	0.142	0.093	0.110	0.161	0.067	0.075	0.063	0.123
TH DA2	0.039	0.105	0.084	0.213	0.114	0.057	0.073	0.050	0.067	0.051	0.112	0.169	0.099	0.117	0.160	0.095	0.094	0.098	0.117
IN DEL	0.092	0.158	0.074	0.178	0.149	0.084	0.130	0.124	0.112	0.133	0.088	0.160	0.106	0.122	0.067	0.118	0.109	0.120	0.000
PH BUS	0.093	0.153	0.069	0.238	0.178	0.096	0.140	0.127	0.101	0.153	0.086	0.150	0.104	0.116	0.063	0.118	0.113	0.116	0.027
PH GUG	0.132	0.213	0.140	0.271	0.197	0.165	0.160	0.165	0.146	0.175	0.145	0.213	0.158	0.184	0.160	0.178	0.165	0.158	0.110
PH GUZ	0.051	0.112	0.057	0.159	0.110	0.071	0.088	0.089	0.084	0.092	0.072	0.143	0.084	0.102	0.068	0.079	0.077	0.082	0.027
PH LU1	0.125	0.221	0.161	0.332	0.220	0.140	0.176	0.170	0.142	0.183	0.154	0.228	0.150	0.158	0.098	0.191	0.169	0.176	0.069
PH LU2	0.067	0.137	0.081	0.219	0.137	0.086	0.106	0.112	0.092	0.117	0.099	0.159	0.111	0.130	0.080	0.136	0.129	0.127	0.039
PH LU3	0.034	0.113	0.059	0.181	0.089	0.055	0.064	0.071	0.062	0.070	0.084	0.127	0.082	0.108	0.103	0.091	0.089	0.091	0.066
PH MIN	0.032	0.126	0.072	0.174	0.102	0.056	0.077	0.089	0.079	0.081	0.081	0.156	0.081	0.100	0.089	0.089	0.087	0.088	0.066
PH PA1	0.059	0.165	0.054	0.227	0.141	0.078	0.106	0.113	0.093	0.127	0.088	0.169	0.121	0.136	0.099	0.116	0.102	0.100	0.012
PH PA2	0.095	0.193	0.114	0.257	0.183	0.132	0.151	0.156	0.136	0.169	0.130	0.195	0.163	0.179	0.116	0.157	0.144	0.142	0.042
PH PA3	0.055	0.152	0.087	0.221	0.158	0.091	0.110	0.115	0.097	0.124	0.088	0.159	0.094	0.124	0.072	0.128	0.118	0.120	0.017
PH PA4	0.069	0.153	0.072	0.209	0.142	0.093	0.108	0.113	0.099	0.120	0.094	0.173	0.107	0.123	0.058	0.116	0.106	0.104	0.030
PH PA5	0.079	0.155	0.085	0.233	0.145	0.104	0.125	0.129	0.114	0.135	0.104	0.157	0.134	0.148	0.103	0.113	0.106	0.112	0.039
MY PEN	0.052	0.152	0.049	0.196	0.134	0.079	0.110	0.107	0.100	0.103	0.073	0.144	0.125	0.135	0.151	0.020	0.014	0.018	0.110

Table S4. Continued.

	TH BAN	TH CHA	TH DA1	TH DA2	IN DEL	PH BUS	PH GUG	PH GUZ	PH LU1	PH LU2	PH LU3	PH MIN	PH PA1	PH PA2	PH PA3	PH PA4	PH PA5	MY PEN
KR 001																		
KR 002																		
KR 003																		
KR 004																		
TW CHU																		
TW JIA																		
TW KAO																		
TW TIC																		
TW TIN																		
TW TIP																		
CN FJS																		
CN YUN																		
VN BC1																		
VN BC2																		
VN HCM																		
LA VI1																		
LA VI2																		
LA VI3																		
MM YAN																		
TH BAN	0.000																	
TH CHA	0.025	0.000																
TH DA1	0.044	0.049	0.000															
TH DA2	0.034	0.043	0.032	0.000														
IN DEL	0.109	0.107	0.123	0.111	0.000													
PH BUS	0.126	0.135	0.120	0.112	0.042	0.000												
PH GUG	0.161	0.174	0.162	0.161	0.114	0.124	0.000											
PH GUZ	0.070	0.080	0.076	0.079	0.029	-0.005	0.095	0.000										
PH LU1	0.137	0.175	0.165	0.139	0.076	0.031	0.132	0.048	0.000									
PH LU2	0.082	0.101	0.104	0.104	0.037	-0.011	0.068	0.004	0.014	0.000								
PH LU3	0.061	0.077	0.067	0.069	0.068	0.029	0.067	0.018	0.048	0.014	0.000							
PH MIN	0.047	0.086	0.073	0.068	0.065	0.043	0.103	-0.015	0.038	0.009	0.015	0.000						
PH PA1	0.090	0.110	0.113	0.101	0.038	0.011	0.054	0.004	0.039	-0.011	0.015	0.022	0.000					
PH PA2	0.138	0.155	0.151	0.147	0.060	0.049	0.046	0.047	0.058	0.016	0.054	0.063	-0.021	0.000				
PH PA3	0.098	0.119	0.104	0.087	0.020	0.018	0.090	-0.006	0.031	-0.011	0.025	0.006	-0.015	0.012	0.000			
PH PA4	0.081	0.109	0.106	0.097	0.038	-0.009	0.040	-0.005	0.037	-0.018	0.020	0.015	-0.017	0.005	0.004	0.000		
PH PA5	0.116	0.138	0.126	0.120	0.055	0.006	0.079	0.018	0.047	0.014	0.036	0.038	-0.010	0.010	0.018	0.001	0.000	
MY PEN	0.105	0.108	0.080	0.100	0.122	0.125	0.172	0.083	0.164	0.134	0.095	0.080	0.106	0.146	0.120	0.115	0.117	0.000

Table S5. Table output of the Evanno et al. [83] method results. The largest value in the *Delta K* column is shown on raw *K*=2.

K	Reps	Mean LnP(K)	Stdev LnP(K)	Ln'(K)	|Ln''(K)|	*Delta K*
1	5	-35633.72	0.545	NA	NA	NA
2	5	-33452.74	2.5393	2180.98	805.78	317.324767
3	5	-32077.54	3.5865	1375.2	669.7	186.727879
4	5	-31372.04	118.5149	705.5	56.52	0.476902
5	5	-30723.06	203.1494	648.98	214.3	1.054889
6	5	-30288.38	5.2184	434.68	163.02	31.239287
7	5	-30016.72	42.8066	271.66	18.1	0.422832
8	5	-29726.96	59.0658	289.76	150.34	2.545297
9	5	-29587.54	46.3758	139.42	89.82	1.936785
10	5	-29537.94	392.4457	49.6	259.26	0.660626
11	5	-29229.08	118.0976	308.86	203.76	1.725353
12	5	-29123.98	209.3351	105.1	265.18	1.266773
13	5	-29284.06	611.9755	-160.08	461.66	0.754377
14	5	-28982.48	113.2591	301.58	134.98	1.191781
15	5	-28815.88	113.5653	166.6	NA	NA

Table S6. Mean assignment rate of *B. dorsalis* individuals into (rows) and from (columns) each population using GeneClass 2 [86]. Values in bold indicate the proportions of individuals assigned to the source population. Zero values were excluded from the table.

	KR 001	KR 002	KR 003	KR 004	TW CHU	TW JIA	TW KAO	TW TIC	TW TIN	TW TIP	CN FJS	CN SHA	CN YUN	VN BC1	VN BC2	VN HCM	LA VI1	LA VI2	LA VI3	MM YAN
KR 001	**0.815**	0.001	0.140		0.005	0.301	0.325	0.210	0.342	0.108	0.004			0.015	0.026	0.002	0.013	0.026	0.050	0.020
KR 002	0.049	**0.860**	0.050		0.001	0.153	0.150	0.134	0.139	0.034				0.008	0.002			0.001	0.010	0.001
KR 003	0.003		**0.851**			0.074	0.021	0.024	0.061	0.011	0.033		0.001	0.011	0.017	0.001	0.124	0.089	0.122	0.052
KR 004	0.005		0.217	**1.000**		0.043	0.001	0.002	0.019		0.239		0.001	0.048	0.026	0.014	0.048	0.008	0.034	0.011
TW CHU	0.078		0.160		**0.785**	0.321	0.437	0.220	0.247	0.133	0.001			0.007	0.005		0.001	0.004	0.018	0.003
TW JIA	0.015		0.053		0.002	**0.789**	0.026	0.086	0.086	0.038				0.001	0.002			0.001	0.006	
TW KAO	0.068	0.003	0.074		0.018	0.185	**0.698**	0.280	0.221	0.129	0.002			0.006	0.009	0.001	0.001	0.002	0.012	0.002
TW TIC	0.037		0.040		0.003	0.158	0.163	**0.672**	0.206	0.174				0.001	0.002	0.001		0.002	0.007	0.001
TW TIN	0.043	0.001	0.090		0.007	0.185	0.241	0.266	**0.663**	0.154				0.007	0.010		0.001	0.004	0.014	0.002
TW TIP	0.038		0.061		0.007	0.203	0.252	0.420	0.209	**0.739**				0.001	0.003		0.001	0.003	0.012	0.001
CN FJS	0.033		0.241		0.001	0.071	0.039	0.022	0.056	0.011	**0.576**	0.027	0.010	0.043	0.037	0.011	0.062	0.066	0.063	0.051
CN SHA			0.006			0.004		0.002				**1.000**		0.001	0.001	0.005			0.001	0.007
CN YUN	0.001		0.197			0.075	0.006	0.010	0.024	0.007	0.012		**0.620**	0.107	0.122	0.002	0.009	0.019	0.023	0.012
VN BC1	0.005		0.074			0.076	0.008	0.010	0.019	0.004	0.005	0.079	0.003	**0.616**	0.129	0.003	0.001	0.007	0.009	0.005
VN BC2	0.007		0.143		0.001	0.095	0.009	0.011	0.019	0.006	0.008	0.177	0.010	0.239	**0.617**	0.006	0.002	0.012	0.010	0.007
VN HCM	0.001		0.025			0.013		0.001	0.001			0.057		0.007	0.002	**0.649**		0.005	0.009	0.129
LA VI1	0.045		0.255		0.005	0.144	0.035	0.068	0.101	0.045	0.052	0.001	0.003	0.022	0.024	0.007	**0.615**	0.189	0.231	0.033
LA VI2	0.074		0.253		0.001	0.151	0.037	0.044	0.087	0.041	0.062		0.002	0.011	0.031	0.025	0.276	**0.604**	0.318	0.045
LA VI3	0.027		0.215		0.001	0.136	0.024	0.037	0.075	0.033	0.043	0.001	0.004	0.012	0.016	0.012	0.226	0.230	**0.604**	0.039
MM YAN	0.002		0.079			0.023	0.001	0.002	0.012		0.005	0.034		0.004	0.005	0.032	0.010	0.013	0.021	**0.609**
TH BAN	0.054		0.020			0.084	0.021	0.064	0.119	0.045				0.001	0.001			0.001	0.008	0.001
TH CHA	0.003		0.008			0.054	0.003	0.049	0.037	0.016									0.006	
TH DA1	0.134	0.003	0.073		0.004	0.145	0.124	0.286	0.299	0.230	0.002	0.022	0.001	0.013	0.007	0.002	0.029	0.024	0.038	0.004
TH DA2	0.070		0.027			0.094	0.053	0.160	0.087	0.096				0.009	0.005			0.004	0.007	0.001
KH PNP			0.201			0.005			0.010		0.001		0.001	0.006	0.007	0.038	0.001	0.004	0.020	0.860
IN DEL	0.001		0.066			0.030		0.002	0.004	0.001		0.031		0.014	0.007	0.028		0.004	0.011	0.201
NP POK			0.041			0.018			0.001			0.016				0.012			0.004	0.050
PH BUS	0.001		0.158			0.024	0.002	0.003	0.046	0.002	0.002			0.004	0.004	0.058	0.001	0.003	0.007	0.282
PH GUG	0.006		0.054			0.015	0.005	0.004	0.014	0.001				0.003	0.002	0.005		0.002	0.011	0.055
PH GUZ	0.048		0.066			0.036	0.021	0.016	0.053	0.006	0.002			0.005	0.006	0.038	0.001	0.008	0.013	0.238
PH LU1	0.004		0.115			0.050	0.024	0.028	0.077	0.019	0.003			0.003	0.006	0.177		0.008	0.013	0.351
PH LU2	0.001		0.047			0.022	0.003	0.002	0.017	0.001	0.002			0.002	0.005	0.013		0.003	0.007	0.126
PH LU3	0.040		0.059		0.002	0.066	0.062	0.057	0.101	0.025	0.001			0.003	0.002	0.017	0.001	0.005	0.011	0.083
PH MIN	0.033		0.081			0.072	0.013	0.015	0.041	0.006	0.004	0.001		0.008	0.006	0.018	0.005	0.018	0.018	0.156
PH PA1	0.004		0.085			0.026		0.001	0.012	0.001	0.001			0.003	0.009	0.015	0.001	0.007	0.012	0.309
PH PA2	0.003		0.086			0.019	0.001	0.001	0.011		0.001			0.002	0.002	0.014	0.001	0.005	0.015	0.176
PH PA3	0.038		0.086		0.001	0.045	0.080	0.028	0.074	0.027	0.002			0.021	0.006	0.027	0.002	0.007	0.013	0.209
PH PA4	0.001		0.070			0.010	0.001	0.001	0.007		0.003			0.003	0.002	0.067	0.001	0.004	0.014	0.219
PH PA5	0.009		0.127			0.045	0.004	0.006	0.027	0.003	0.004			0.005	0.006	0.024	0.011	0.019	0.023	0.249
MY PEN	0.050		0.292			0.130	0.024	0.023	0.077	0.021	0.078		0.002	0.011	0.019	0.020	0.320	0.318	0.312	0.047

Table S6. Continued.

	TH BAN	TH CHA	TH DA1	TH DA2	KH PNP	IN DEL	NP POK	PH BUS	PH GUG	PH GUZ	PH LU1	PH LU2	PH LU3	PH MIN	PH PA1	PH PA2	PH PA3	PH PA4	PH PA5	MY PEN
KR 001	0.049	0.043	0.294	0.088		0.009		0.017	0.016	0.119	0.010	0.033	0.208	0.046	0.075	0.003	0.044	0.046	0.003	0.004
KR 002	0.040	0.032	0.150	0.005				0.007	0.001	0.037	0.001	0.004	0.026	0.001	0.005		0.002	0.003		
KR 003	0.002	0.003	0.007	0.001	0.025	0.015		0.020	0.019	0.058	0.003	0.037	0.112	0.057	0.118	0.087	0.018	0.068	0.029	0.075
KR 004			0.004		0.015	0.038		0.042	0.005	0.097	0.002		0.013	0.015	0.006		0.009	0.016		
TW TEP	0.015	0.012	0.047	0.003		0.005		0.006	0.006	0.041	0.003	0.008	0.134	0.006	0.023		0.004	0.005		
TW JIA	0.004	0.007	0.030	0.013				0.004		0.007	0.002	0.001	0.026	0.002	0.007			0.001		
TW KAO	0.018	0.017	0.098	0.004		0.001		0.007	0.007	0.033	0.002	0.011	0.091	0.006	0.021		0.008	0.006		
TW TIC	0.019	0.009	0.112	0.022				0.003	0.001	0.009	0.002	0.002	0.035	0.001	0.007		0.003	0.001		
TW TIN	0.011	0.013	0.121	0.009		0.002		0.006	0.006	0.014	0.005	0.007	0.058	0.002	0.012		0.002	0.004		0.001
TW TIP	0.021	0.025	0.156	0.029		0.001		0.001	0.003	0.012	0.001	0.004	0.048	0.001	0.010		0.001	0.002		
CN FJS	0.002	0.006	0.015	0.002	0.072	0.044	0.048	0.042	0.028	0.099	0.009	0.018	0.119	0.045	0.066	0.020	0.026	0.060	0.024	0.022
CN SHA		0.001				0.022				0.002	0.003		0.004		0.002		0.002			
CN YUN		0.002	0.002		0.056	0.016		0.018	0.010	0.059	0.003	0.007	0.042	0.005	0.016	0.001	0.016	0.014	0.001	0.002
VN BC1	0.004	0.006	0.004	0.001	0.020	0.007	0.002	0.018	0.005	0.023	0.002	0.005	0.019	0.003	0.016		0.015	0.011		
VN BC2	0.004	0.005	0.005	0.004	0.054	0.013	0.019	0.017	0.004	0.042	0.008	0.013	0.019	0.026	0.027		0.007	0.017		0.001
VN HCM	0.001	0.002	0.001		0.087	0.072	0.002	0.089	0.004	0.104	0.019	0.037	0.044	0.027	0.034	0.010	0.039	0.120	0.005	
LA VI1	0.009	0.028	0.047	0.010	0.065	0.030	0.001	0.022	0.010	0.129	0.006	0.018	0.121	0.057	0.071	0.006	0.029	0.063	0.007	0.073
LA VI2	0.012	0.033	0.061	0.013	0.118	0.046	0.001	0.039	0.020	0.163	0.011	0.030	0.178	0.080	0.109	0.020	0.039	0.109	0.020	0.173
LA VI3	0.013	0.029	0.029	0.002	0.107	0.019		0.021	0.010	0.086	0.005	0.011	0.063	0.030	0.058	0.006	0.012	0.051	0.003	0.120
MM YAN	0.001	0.005	0.001		0.385	0.164	0.053	0.043	0.030	0.141	0.021	0.084	0.097	0.037	0.116	0.029	0.046	0.120	0.028	0.004
TH BAN	**0.849**	0.128	0.063	0.045		0.001		0.001	0.001	0.020	0.001	0.018	0.016	0.004	0.014		0.001	0.002		
TH CHA	0.019	**0.896**	0.008	0.002						0.005	0.001	0.003	0.010	0.001	0.001					
TH DA1	0.027	0.044	**0.693**	0.066	0.047	0.003		0.019	0.008	0.046	0.003	0.010	0.098	0.011	0.021		0.013	0.019		0.008
TH DA2	0.032	0.032	0.100	**0.687**	0.001	0.001		0.008	0.006	0.018	0.004	0.009	0.070	0.007	0.022		0.007	0.006		0.001
KH PNP		0.002			**1.000**	0.634	0.503	0.031	0.019	0.347	0.008	0.030	0.030	0.094	0.084	0.050	0.032	0.115	0.071	0.007
IN DEL	0.003	0.004			0.500	**0.739**	0.156	0.016	0.024	0.085	0.007	0.034	0.028	0.030	0.047	0.008	0.045	0.082	0.010	
NP POK					0.890	0.173	**1.000**	0.100	0.022	0.079	0.035	0.033	0.076	0.012	0.061	0.002	0.009	0.083	0.002	
PH BUS	0.001	0.001	0.001	0.003	0.089	0.072	0.252	**0.829**	0.088	0.305	0.157	0.470	0.394	0.116	0.306	0.241	0.207	0.376	0.425	
PH GUG	0.003	0.003	0.006		0.028	0.034	0.064	0.049	**0.630**	0.077	0.017	0.112	0.242	0.045	0.187	0.217	0.076	0.173	0.072	
PH GUZ	0.003	0.007	0.012	0.003	0.350	0.084	0.204	0.115	0.035	**0.777**	0.086	0.199	0.262	0.184	0.193	0.118	0.130	0.231	0.162	0.002
PH_LU1	0.007	0.012	0.011	0.001	0.270	0.178	0.527	0.216	0.104	0.305	**0.754**	0.628	0.505	0.369	0.345	0.198	0.197	0.409	0.165	0.001
PH LU2	0.007	0.020	0.001		0.107	0.042	0.170	0.169	0.095	0.225	0.080	**0.706**	0.272	0.156	0.246	0.178	0.172	0.255	0.145	
PH LU3	0.007	0.013	0.036	0.007	0.034	0.025	0.055	0.075	0.038	0.182	0.055	0.202	**0.609**	0.107	0.144	0.105	0.079	0.126	0.078	0.001
PH MIN	0.012	0.030	0.012	0.012	0.086	0.052	0.077	0.067	0.039	0.309	0.042	0.252	0.318	**0.710**	0.214	0.161	0.170	0.224	0.154	0.006
PH PA1	0.003	0.004	0.001		0.132	0.078	0.135	0.073	0.154	0.226	0.075	0.251	0.322	0.188	**0.818**	0.249	0.173	0.277	0.184	0.002
PH PA2	0.001	0.002	0.001		0.219	0.055	0.030	0.088	0.257	0.223	0.042	0.248	0.287	0.095	0.330	**0.590**	0.146	0.282	0.221	0.001
PH PA3	0.004	0.005	0.033	0.003	0.205	0.099	0.101	0.100	0.059	0.264	0.073	0.315	0.304	0.210	0.248	0.137	**0.788**	0.252	0.158	0.001
PH PA4	0.001	0.003			0.128	0.074		0.137	0.219	0.318	0.055	0.300	0.320	0.125	0.282	0.200	0.097	**0.736**	0.210	0.001
PH PA5	0.003	0.003	0.002	0.001	0.173	0.107	0.148	0.161	0.194	0.345	0.064	0.318	0.395	0.202	0.394	0.348	0.156	0.393	**0.533**	0.005
MY PEN	0.033	0.041	0.040	0.012	0.235	0.023	0.049	0.039	0.028	0.165	0.014	0.036	0.150	0.114	0.143	0.028	0.031	0.120	0.034	**0.590**

Table S7. Allelic frequencies based on the polymorphisms of 15 microsatellite markers at each study site.

	Study sites																				
**Locus**	**Allele/n**	**KR 001**	**KR 002**	**KR 003**	**KR 004**	**TW CHU**	**TW JIA**	**TW KAO**	**TW TIC**	**TW TIN**	**TW TIP**	**CN FJS**	**CN SHA**	**CN YUN**	**VN BC1**	**VN BC2**	**VN HCM**	**LA VI1**	**LA VI2**	**LA VI3**	**MM YAN**
**Bp200**	**N**	9	10	5	2	7	8	20	20	20	15	24	1	24	18	13	18	24	24	24	25
	**94**	0	0	0	0	0	0	0	0	0	0.03333	0	0	0	0	0	0	0	0	0	0
	**98**	0	0	0	0	0	0	0	0	0	0	0	0	0	0	0.03846	0	0	0	0	0
	**100**	0	0	0.1	0	0.42857	0	0.075	0.075	0	0.06667	0.10417	0	0	0	0	0	0.02083	0.02083	0	0
	**101**	0	0	0	0	0	0	0	0	0	0	0	0	0	0.16667	0.07692	0	0	0	0	0
	**102**	0.05556	0	0	0	0	0	0	0.025	0.025	0.03333	0.08333	0	0.04167	0	0.03846	0.05556	0.02083	0.08333	0.0625	0.08
	**103**	0	0	0	0	0	0	0	0	0	0	0	0	0	0.02778	0	0.02778	0	0	0	0
	**104**	0.44444	0.4	0.3	1	0.21429	0.5625	0.6	0.35	0.45	0.56667	0.3125	0	0.33333	0.58333	0.61538	0.52778	0.33333	0.1875	0.3125	0.16
	**106**	0.5	0.5	0.6	0	0.35714	0.4375	0.275	0.375	0.45	0.26667	0.45833	0	0.58333	0.11111	0.15385	0.13889	0.625	0.70833	0.54167	0.66
	**107**	0	0	0	0	0	0	0	0	0.025	0	0	0	0	0.08333	0	0	0	0	0	0
	**108**	0	0	0	0	0	0	0.025	0	0.025	0	0	0	0.04167	0	0	0	0	0	0.0625	0.04
	**109**	0	0	0	0	0	0	0	0	0.025	0	0	0	0	0	0	0	0	0	0	0
	**110**	0	0	0	0	0	0	0.025	0.1	0	0	0.04167	0	0	0	0.07692	0	0	0	0.02083	0.02
	**118**	0	0.1	0	0	0	0	0	0.025	0	0	0	0.5	0	0	0	0	0	0	0	0.02
	**120**	0	0	0	0	0	0	0	0.025	0	0	0	0	0	0	0	0	0	0	0	0
	**122**	0	0	0	0	0	0	0	0	0	0	0	0	0	0	0	0	0	0	0	0
	**124**	0	0	0	0	0	0	0	0	0	0	0	0	0	0	0	0	0	0	0	0
	**126**	0	0	0	0	0	0	0	0.025	0	0.03333	0	0.5	0	0	0	0.25	0	0	0	0.02
	**128**	0	0	0	0	0	0	0	0	0	0	0	0	0	0.02778	0	0	0	0	0	0
**Locus**	**Allele/n**	**KR 001**	**KR 002**	**KR 003**	**KR 004**	**TW CHU**	**TW JIA**	**TW KAO**	**TW TIC**	**TW TIN**	**TW TIP**	**CN FJS**	**CN SHA**	**CN YUN**	**VN BC1**	**VN BC2**	**VN HCM**	**LA VI1**	**LA VI2**	**LA VI3**	**MM YAN**
**Bi4**	**N**	9	10	5	2	7	8	20	20	20	15	24	1	24	18	13	18	24	24	24	25
	**111**	0	0	0	0	0	0.125	0	0	0	0	0	0	0	0	0.03846	0	0	0	0	0
	**112**	0	0	0	0	0	0	0	0.05	0	0	0	0	0	0	0	0	0	0	0	0
	**114**	0	0	0	0	0	0	0	0	0	0	0	0	0	0	0	0	0	0	0	0
	**115**	0	0	0	0	0	0	0	0.025	0	0	0	0	0	0	0	0	0	0	0	0
	**120**	0	0	0	0	0	0.0625	0	0.025	0.05	0	0	0	0	0	0	0	0	0	0	0
	**122**	0	0	0	0	0	0	0	0.05	0	0	0	0	0	0	0	0	0	0	0	0
	**123**	0	0	0	0	0	0.125	0	0.025	0	0	0	0	0	0	0	0	0	0	0	0
	**124**	0	0	0.2	0	0	0	0	0.025	0	0.06667	0.20833	0	0.08333	0	0	0	0.10417	0	0.125	0.04
	**125**	0.22222	0.5	0.1	0	0.28571	0.125	0.225	0.05	0.175	0.03333	0	0	0.04167	0	0.03846	0	0	0	0	0.14
	**126**	0.61111	0.5	0.5	1	0.64286	0.4375	0.675	0.375	0.55	0.66667	0.60417	1	0.75	0.97222	0.80769	1	0.64583	0.85417	0.6875	0.72
	**127**	0	0	0	0	0	0	0	0	0	0	0	0	0	0	0	0	0.04167	0.02083	0	0
	**128**	0.16667	0	0.2	0	0.07143	0.125	0.1	0.25	0.225	0.2	0.1875	0	0.125	0.02778	0.07692	0	0.20833	0.125	0.1875	0.1
	**129**	0	0	0	0	0	0	0	0.025	0	0	0	0	0	0	0	0	0	0	0	0
	**130**	0	0	0	0	0	0	0	0.025	0	0.03333	0	0	0	0	0	0	0	0	0	0
	**133**	0	0	0	0	0	0	0	0	0	0	0	0	0	0	0	0	0	0	0	0
	**134**	0	0	0	0	0	0	0	0.05	0	0	0	0	0	0	0.03846	0	0	0	0	0
	**136**	0	0	0	0	0	0	0	0	0	0	0	0	0	0	0	0	0	0	0	0
	**139**	0	0	0	0	0	0	0	0.025	0	0	0	0	0	0	0	0	0	0	0	0

Table S7. Continued.

	Study sites																				
**Locus**	**Allele/n**	**KR 001**	**KR 002**	**KR 003**	**KR 004**	**TW CHU**	**TW JIA**	**TW KAO**	**TW TIC**	**TW TIN**	**TW TIP**	**CN FJS**	**CN SHA**	**CN YUN**	**VN BC1**	**VN BC2**	**VN HCM**	**LA VI1**	**LA VI2**	**LA VI3**	**MM YAN**
**Bt5.8a**	**N**	9	10	5	2	7	8	20	20	20	15	24	1	24	18	13	18	24	24	24	25
	**101**	0	0	0	0	0	0	0	0	0	0	0	0	0	0	0	0	0	0	0	0
	**105**	0	0	0	0	0	0	0	0	0	0	0	0	0	0	0	0	0	0	0	0
	**106**	0	0	0	0	0	0	0	0	0	0	0	0	0.02083	0	0	0	0	0	0	0
	**107**	0	0	0	0	0	0	0	0.025	0	0	0	0	0	0	0	0	0	0	0	0
	**109**	0	0	0	0	0	0	0	0.025	0	0	0	0	0	0.02778	0	0	0	0	0	0
	**111**	0	0	0	0	0	0	0	0.05	0.05	0	0	0	0	0	0	0	0	0	0	0.02
	**112**	0	0	0	0	0	0	0	0	0	0	0	0	0	0	0.03846	0	0	0	0	0
	**113**	0	0	0	0	0	0	0	0.025	0	0	0	0	0	0	0	0	0	0	0	0
	**114**	0	0	0	0	0	0	0	0	0	0	0	0	0	0	0	0	0	0	0	0
	**115**	0.05556	0	0	0	0.07143	0	0.025	0	0	0	0	0	0	0	0	0	0.10417	0.08333	0.08333	0
	**116**	0	0	0	0	0	0	0	0	0	0	0	0	0.04167	0.02778	0.03846	0	0	0	0	0
	**117**	0	0	0	0	0	0	0	0	0	0	0	0	0	0	0.03846	0	0	0	0	0
	**118**	0	0	0	0	0	0	0	0	0	0	0	0	0	0.02778	0	0	0	0	0	0
	**119**	0	0	0	0	0	0	0	0.025	0	0.03333	0	0	0	0	0	0	0	0	0	0
	**120**	0	0	0	0	0	0	0	0	0	0	0	0	0	0	0	0.05556	0	0	0	0
	**121**	0.27778	0.25	0	0	0	0.0625	0.275	0.175	0	0.33333	0	0	0.10417	0.05556	0.11538	0	0	0	0	0
	**122**	0	0	0.4	0	0	0	0	0	0	0	0.1875	0	0	0	0	0	0.125	0.1875	0.1875	0.1
	**123**	0	0	0	0	0	0	0	0	0	0	0	0	0	0	0	0	0	0	0	0
	**124**	0	0	0	0	0	0	0	0	0.025	0	0	0	0	0	0	0	0	0.02083	0.04167	0
	**125**	0	0.05	0	0	0	0	0	0	0	0	0	0	0	0	0	0	0	0	0	0
	**126**	0	0	0	0	0	0	0	0	0	0	0	0	0	0.05556	0	0	0	0	0	0
	**127**	0	0	0	0	0	0	0	0	0	0	0	0	0	0	0	0	0	0.02083	0	0
	**128**	0.44444	0.7	0.4	1	0.85714	0.5625	0.575	0.4	0.65	0.46667	0.75	0.5	0.77083	0.80556	0.69231	0.41667	0.64583	0.5625	0.58333	0.36
	**130**	0.22222	0	0.2	0	0.07143	0.1875	0.075	0.05	0.125	0.13333	0.04167	0	0.0625	0	0.07692	0.02778	0.10417	0.04167	0.08333	0.02
	**132**	0	0	0	0	0	0	0	0	0	0	0	0	0	0	0	0	0	0.02083	0	0.02
	**133**	0	0	0	0	0	0	0.025	0.05	0.05	0	0	0	0	0	0	0	0	0	0	0
	**134**	0	0	0	0	0	0	0	0	0	0	0	0	0	0	0	0	0	0.02083	0	0
	**137**	0	0	0	0	0	0	0	0	0	0	0	0	0	0	0	0	0	0	0	0
	**138**	0	0	0	0	0	0.0625	0	0.05	0	0.03333	0	0	0	0	0	0	0	0	0	0
	**139**	0	0	0	0	0	0	0.025	0.05	0.05	0	0.02083	0	0	0	0	0.02778	0.02083	0.02083	0.02083	0
	**144**	0	0	0	0	0	0	0	0.05	0	0	0	0	0	0	0	0	0	0.02083	0	0
	**150**	0	0	0	0	0	0	0	0	0	0	0	0	0	0	0	0.02778	0	0	0	0
	**151**	0	0	0	0	0	0	0	0.025	0	0	0	0	0	0	0	0	0	0	0	0
	**152**	0	0	0	0	0	0	0	0	0	0	0	0.5	0	0	0	0.44444	0	0	0	0.48
	**157**	0	0	0	0	0	0.125	0	0	0	0	0	0	0	0	0	0	0	0	0	0
	**160**	0	0	0	0	0	0	0	0	0.05	0	0	0	0	0	0	0	0	0	0	0

Table S7. Continued.

	Study sites																				
**Locus**	**Allele/n**	**KR 001**	**KR 002**	**KR 003**	**KR 004**	**TW CHU**	**TW JIA**	**TW KAO**	**TW TIC**	**TW TIN**	**TW TIP**	**CN FJS**	**CN SHA**	**CN YUN**	**VN BC1**	**VN BC2**	**VN HCM**	**LA VI1**	**LA VI2**	**LA VI3**	**MM YAN**
**Bt4**	N	9	10	5	2	7	8	20	20	20	15	24	1	24	18	13	18	24	24	24	25
	**100**	0	0	0	0	0	0	0	0	0	0.06667	0	0	0	0	0	0	0	0	0	0
	**102**	0	0	0	0	0	0	0	0	0	0	0	0	0	0.13889	0	0	0	0	0	0
	**103**	0	0	0	0	0	0	0	0	0	0	0	0	0	0	0	0	0	0	0.04167	0.02
	**104**	0	0	0	0	0	0	0	0	0	0	0	0	0	0.02778	0	0	0	0	0	0
	**107**	0	0	0	0	0	0.0625	0	0	0	0	0	0	0	0	0	0	0	0	0	0
	**113**	0	0	0	0	0	0	0	0.025	0	0	0	0	0	0	0	0	0	0	0	0
	**115**	0	0	0.1	0	0	0	0	0.05	0.05	0.03333	0.04167	0	0.02083	0	0	0	0	0.0625	0.02083	0.02
	**116**	0.05556	0.2	0	0	0	0.0625	0.025	0	0	0	0	0	0.04167	0.05556	0	0	0	0	0	0
	**117**	0	0	0	0	0	0	0	0.025	0	0	0	0	0	0	0	0	0	0	0	0
	**118**	0	0	0	0	0	0	0.025	0	0.075	0	0	0	0	0.02778	0	0.05556	0.04167	0.0625	0	0.12
	**119**	0	0	0	0	0	0	0	0	0.025	0	0	0	0	0	0	0	0	0	0	0
	**120**	0	0	0.1	0	0	0.0625	0.075	0	0.05	0.03333	0.04167	0	0.0625	0	0	0.02778	0.04167	0	0.02083	0.04
	**121**	0	0	0	0	0	0	0	0	0	0	0	0	0.02083	0	0	0	0	0	0	0
	**122**	0.16667	0.1	0.1	0.5	0.28571	0.125	0.1	0.125	0.05	0	0.0625	0.5	0.02083	0.11111	0.19231	0.13889	0.10417	0.0625	0.08333	0.04
	**124**	0.22222	0	0.2	0	0.5	0.1875	0.325	0.25	0.325	0.23333	0.16667	0	0.33333	0.11111	0.23077	0.44444	0.41667	0.5	0.54167	0.34
	**126**	0.22222	0.05	0.2	0	0.21429	0.1875	0.125	0.125	0.125	0.3	0.22917	0	0.27083	0.25	0.23077	0.19444	0.10417	0.20833	0.22917	0.3
	**128**	0.05556	0	0	0.5	0	0	0.125	0.175	0.075	0.13333	0.29167	0	0.10417	0.05556	0	0.11111	0.125	0.04167	0	0.02
	**130**	0.11111	0.15	0.1	0	0	0.125	0.075	0.025	0.1	0.06667	0.0625	0	0.02083	0.08333	0.03846	0	0.08333	0.04167	0.02083	0.04
	**132**	0.16667	0.5	0.2	0	0	0.0625	0.075	0.15	0.1	0	0.10417	0.5	0.08333	0.05556	0.15385	0	0.0625	0.02083	0.04167	0.04
	**133**	0	0	0	0	0	0	0	0	0	0	0	0	0	0	0.03846	0	0	0	0	0
	**134**	0	0	0	0	0	0.125	0.025	0.025	0.025	0.03333	0	0	0	0.02778	0	0	0	0	0	0.02
	**135**	0	0	0	0	0	0	0	0	0	0	0	0	0	0	0	0	0	0	0	0
	**136**	0	0	0	0	0	0	0	0	0	0	0	0	0	0.02778	0	0	0	0	0	0
	**137**	0	0	0	0	0	0	0	0	0	0	0	0	0	0	0	0	0.02083	0	0	0
	**139**	0	0	0	0	0	0	0.025	0.025	0	0.03333	0	0	0.02083	0	0.03846	0	0	0	0	0
	**140**	0	0	0	0	0	0	0	0	0	0	0	0	0	0	0	0.02778	0	0	0	0
	**141**	0	0	0	0	0	0	0	0	0	0	0	0	0	0.02778	0	0	0	0	0	0
	**146**	0	0	0	0	0	0	0	0	0	0	0	0	0	0	0.03846	0	0	0	0	0
	**148**	0	0	0	0	0	0	0	0	0	0.06667	0	0	0	0	0	0	0	0	0	0
	**156**	0	0	0	0	0	0	0	0	0	0	0	0	0	0	0.03846	0	0	0	0	0

Table S7. Continued.

	Study sites																				
**Locus**	**Allele/n**	**KR 001**	**KR 002**	**KR 003**	**KR 004**	**TW CHU**	**TW JIA**	**TW KAO**	**TW TIC**	**TW TIN**	**TW TIP**	**CN FJS**	**CN SHA**	**CN YUN**	**VN BC1**	**VN BC2**	**VN HCM**	**LA VI1**	**LA VI2**	**LA VI3**	**MM YAN**
**Bi8**	N	8	10	5	2	7	8	20	20	20	15	24	1	24	18	13	18	24	24	24	25
	**99**	0	0	0	0	0	0	0	0	0	0	0	0	0	0	0	0	0	0	0.02083	0
	**104**	0	0	0	0	0	0	0	0	0	0	0	0	0	0	0	0.02778	0	0	0	0
	**110**	0	0	0	0	0	0	0	0	0	0	0	0	0	0	0	0	0	0	0.02083	0
	**113**	0	0	0	0	0	0	0	0	0.05	0	0	0	0	0	0	0	0	0	0	0
	**114**	0	0	0	0	0	0	0	0	0	0	0	0	0	0	0	0	0	0	0.02083	0
	**117**	0.0625	0	0	0	0	0	0	0	0.025	0	0	0	0	0	0	0	0	0	0	0
	**120**	0.0625	0.05	0	0	0	0.125	0	0	0	0	0	0	0.0625	0	0	0.16667	0.04167	0.10417	0	0.04
	**121**	0	0	0	0	0	0	0	0	0	0	0	0	0	0	0	0	0	0	0	0
	**123**	0	0.1	0	0	0	0	0	0	0	0.03333	0	0	0	0	0	0	0	0	0	0
	**124**	0	0	0	0	0.07143	0	0.025	0.05	0	0	0	0	0	0	0	0	0	0.02083	0	0
	**125**	0	0	0	0	0	0	0	0	0	0	0	0	0	0	0	0	0	0	0	0
	**127**	0	0	0	0	0.14286	0.125	0.075	0.075	0	0	0	0	0	0	0	0	0	0	0	0
	**129**	0.0625	0	0	0	0	0	0	0	0	0	0	0	0	0	0	0	0	0	0	0
	**130**	0	0	0	0	0	0	0	0	0.025	0	0	0	0	0	0	0	0	0	0	0
	**131**	0	0.2	0.1	0	0.07143	0	0.025	0.05	0.075	0.06667	0.125	0	0	0	0	0.08333	0.10417	0.02083	0.125	0.06
	**132**	0	0	0	0	0	0	0	0	0	0	0	0	0.25	0	0.03846	0	0	0	0	0
	**133**	0	0	0	0	0.07143	0.0625	0.05	0.05	0.05	0.06667	0.08333	0	0.0625	0.11111	0	0.05556	0.08333	0.08333	0.125	0.18
	**135**	0.375	0.05	0.3	0	0.07143	0.125	0.1	0.125	0.175	0.13333	0.33333	0	0.02083	0	0.03846	0.47222	0.20833	0.52083	0.35417	0.38
	**136**	0	0	0	0	0	0.0625	0.1	0.025	0.05	0.1	0	0	0.4375	0.25	0.61538	0	0	0	0	0
	**137**	0.125	0.25	0	0	0	0	0.05	0.025	0.025	0.03333	0.04167	1	0	0.13889	0	0	0.08333	0	0	0.08
	**138**	0	0	0	0	0	0	0	0	0	0	0	0	0.02083	0.02778	0	0	0	0	0	0
	**139**	0.1875	0.15	0.5	1	0.35714	0.3125	0.325	0.375	0.225	0.36667	0.375	0	0.14583	0.44444	0.30769	0.16667	0.47917	0.25	0.33333	0.26
	**140**	0	0	0	0	0	0	0.025	0.025	0.025	0.03333	0	0	0	0.02778	0	0	0	0	0	0
	**141**	0	0.1	0	0	0.07143	0.0625	0.1	0.1	0.025	0.03333	0	0	0	0	0	0.02778	0	0	0	0
	**143**	0.0625	0.05	0.1	0	0.07143	0.0625	0.025	0	0.025	0.06667	0	0	0	0	0	0	0	0	0	0
	**144**	0	0	0	0	0	0	0.05	0.05	0.05	0	0.04167	0	0	0	0	0	0	0	0	0
	**145**	0	0.05	0	0	0	0	0	0	0.025	0	0	0	0	0	0	0	0	0	0	0
	**146**	0	0	0	0	0	0	0	0	0.05	0	0	0	0	0	0	0	0	0	0	0
	**147**	0.0625	0	0	0	0.07143	0.0625	0	0	0.05	0	0	0	0	0	0	0	0	0	0	0
	**149**	0	0	0	0	0	0	0.05	0.05	0.05	0.06667	0	0	0	0	0	0	0	0	0	0

Table S7. Continued.

	Study sites																				
**Locus**	**Allele/n**	**KR 001**	**KR 002**	**KR 003**	**KR 004**	**TW CHU**	**TW JIA**	**TW KAO**	**TW TIC**	**TW TIN**	**TW TIP**	**CN FJS**	**CN SHA**	**CN YUN**	**VN BC1**	**VN BC2**	**VN HCM**	**LA VI1**	**LA VI2**	**LA VI3**	**MM YAN**
**Bt6.8a**	**N**	9	10	5	2	7	8	20	20	20	15	24	1	24	18	13	18	24	24	24	25
	**103**	0	0	0	0	0	0.0625	0	0	0	0	0	0	0	0	0	0	0	0	0	0
	**104**	0	0	0	0	0	0	0	0	0	0	0	0	0	0	0	0.02778	0	0	0	0
	**109**	0	0	0	0	0	0.125	0	0	0	0	0	0	0	0	0	0	0	0	0	0
	**111**	0	0	0.2	0	0	0	0	0	0	0	0	0	0	0	0	0	0	0	0	0.04
	**117**	0	0	0	0	0	0	0	0	0	0	0	0	0	0	0	0	0	0	0.02083	0
	**118**	0	0	0	0	0	0	0	0	0	0	0	0	0	0	0	0.02778	0	0	0	0
	**119**	0	0	0	0	0	0	0	0	0	0	0	0	0	0	0	0	0	0	0	0
	**132**	0	0	0	0	0	0	0	0	0	0	0	0	0	0	0	0	0	0	0	0
	**133**	0	0	0	0	0	0	0	0	0	0	0	0	0	0	0	0	0	0	0	0
	**135**	0	0	0	0	0	0	0	0	0	0.06667	0	0	0	0	0	0	0	0.02083	0	0.1
	**137**	0	0	0	0	0	0	0	0	0	0	0	0	0	0	0	0	0.02083	0	0	0
	**140**	0	0	0	0	0	0	0	0	0	0	0	0	0	0	0	0	0.04167	0.04167	0.10417	0.02
	**141**	0	0	0	0	0	0.0625	0	0	0.025	0	0	0	0	0	0	0	0	0	0	0
	**142**	0	0	0	0	0	0	0	0	0	0	0	0	0	0.05556	0	0	0	0	0	0
	**144**	0.11111	0.15	0	0.5	0.28571	0.125	0.125	0.225	0.225	0.06667	0.25	0	0.41667	0.16667	0.03846	0	0.22917	0.10417	0.10417	0.22
	**146**	0	0	0	0	0	0	0	0	0	0	0	0	0	0	0	0	0	0	0.0625	0.02
	**147**	0	0	0	0	0	0	0	0	0	0	0	0	0	0	0	0	0	0	0	0
	**150**	0.27778	0	0	0	0	0	0	0	0.05	0	0	0	0	0.19444	0.03846	0	0	0.02083	0	0
	**151**	0	0	0	0	0	0	0	0	0	0	0	0.5	0	0	0.03846	0.13889	0.02083	0.08333	0.04167	0
	**153**	0.11111	0.65	0	0	0.28571	0.0625	0.15	0.025	0.075	0.03333	0	0	0	0	0	0	0	0	0.08333	0
	**154**	0	0	0.1	0	0	0.0625	0	0	0.025	0.03333	0.04167	0	0.20833	0.22222	0.15385	0.36111	0.16667	0.1875	0.1875	0.18
	**156**	0	0	0.1	0	0	0	0.025	0.075	0.025	0.03333	0.04167	0	0	0	0	0	0	0	0	0.02
	**157**	0	0	0.1	0	0	0	0	0	0	0	0.08333	0	0.02083	0.02778	0.03846	0	0.04167	0	0	0
	**159**	0.38889	0	0.2	0.5	0	0.125	0.125	0.1	0.125	0.13333	0.1875	0.5	0.20833	0.25	0.5	0.44444	0.33333	0.45833	0.29167	0.36
	**161**	0	0	0	0	0	0	0	0	0	0	0	0	0	0	0	0	0	0	0	0.02
	**162**	0	0	0	0	0	0	0	0	0	0	0	0	0	0	0	0	0.02083	0	0	0
	**168**	0.11111	0.2	0.1	0	0.42857	0.25	0.575	0.55	0.4	0.56667	0	0	0	0	0.03846	0	0	0	0	0
	**169**	0	0	0.2	0	0	0.0625	0	0	0	0	0.39583	0	0.10417	0.05556	0.11538	0	0.08333	0.04167	0.02083	0.02
	**171**	0	0	0	0	0	0	0	0	0	0	0	0	0	0	0	0	0	0	0	0
	**173**	0	0	0	0	0	0	0	0	0	0	0	0	0	0	0	0	0	0	0.02083	0
	**174**	0	0	0	0	0	0	0	0.025	0	0.06667	0	0	0.04167	0	0	0	0.02083	0.02083	0.02083	0
	**175**	0	0	0	0	0	0	0	0	0	0	0	0	0	0	0	0	0	0	0	0
	**178**	0	0	0	0	0	0	0	0	0	0	0	0	0	0	0	0	0	0	0.02083	0
	**181**	0	0	0	0	0	0	0	0	0.025	0	0	0	0	0	0	0	0	0	0	0
	**182**	0	0	0	0	0	0	0	0	0	0	0	0	0	0	0.03846	0	0	0	0	0
	**184**	0	0	0	0	0	0	0	0	0	0	0	0	0	0.02778	0	0	0	0	0.02083	0
	**186**	0	0	0	0	0	0.0625	0	0	0.025	0	0	0	0	0	0	0	0	0	0	0
	**189**	0	0	0	0	0	0	0	0	0	0	0	0	0	0	0	0	0.02083	0.02083	0	0
	**190**	0	0	0	0	0	0	0	0	0	0	0	0	0	0	0	0	0	0	0	0

Table S7. Continued.

	Study sites																				
**Locus**	**Allele/n**	**KR 001**	**KR 002**	**KR 003**	**KR 004**	**TW CHU**	**TW JIA**	**TW KAO**	**TW TIC**	**TW TIN**	**TW TIP**	**CN FJS**	**CN SHA**	**CN YUN**	**VN BC1**	**VN BC2**	**VN HCM**	**LA VI1**	**LA VI2**	**LA VI3**	**MM YAN**
**Boms3a**	**N**	8	6	5	2	7	8	20	20	20	15	24	1	24	18	13	18	24	24	24	25
	**102**	0	0.08333	0	0	0	0	0.05	0.05	0.025	0.03333	0	0	0	0	0	0	0	0	0	0
	**106**	0	0	0	0	0	0	0	0	0.05	0.06667	0	0	0	0	0	0	0	0	0	0
	**108**	0	0	0	0	0	0	0	0	0	0	0	0	0	0	0	0	0	0	0	0.02
	**110**	0	0	0	0	0	0.125	0.025	0.025	0	0	0	0	0	0	0	0	0	0	0	0
	**115**	0	0	0	0	0	0	0	0	0.025	0	0	0	0	0	0	0	0	0	0	0
	**117**	0	0	0	0	0	0	0	0.025	0	0	0	0	0	0	0	0.02778	0	0	0	0
	**120**	0	0	0	0	0	0	0	0	0	0	0.02083	0	0	0	0	0	0	0	0	0
	**124**	0	0	0	0	0	0	0	0	0	0	0	0.5	0	0	0	0	0	0	0	0
	**126**	0	0	0	0	0	0	0.025	0.025	0	0	0	0	0	0	0	0	0	0	0	0
	**133**	0	0	0	0	0	0	0.025	0.025	0	0	0	0	0	0	0	0	0	0	0	0
	**134**	0	0	0	0	0	0	0.025	0	0	0.03333	0	0	0	0	0	0	0	0	0	0
	**135**	0	0	0	0	0	0	0	0	0.025	0.03333	0.04167	0	0	0	0	0	0	0	0.0625	0
	**136**	0	0	0	0	0	0	0.025	0.025	0.025	0.03333	0	0	0	0	0	0	0	0	0	0
	**138**	0	0	0	0	0	0	0	0	0.025	0	0	0	0	0	0	0	0	0	0	0
	**139**	0	0	0.1	0	0	0	0	0	0	0	0.02083	0	0	0.11111	0.03846	0.05556	0	0	0.04167	0.02
	**140**	0	0	0	0	0	0	0.05	0.025	0.05	0.06667	0	0	0	0	0	0	0	0	0	0
	**142**	0	0	0	0	0.14286	0.125	0	0	0	0	0	0	0	0	0	0	0	0	0	0
	**144**	0	0	0	0	0.14286	0.125	0	0	0	0	0	0	0	0	0	0	0	0	0	0.02
	**147**	0	0	0	0	0	0	0	0	0	0	0	0	0	0	0	0	0	0	0	0
	**150**	0	0	0	0	0	0	0	0	0	0	0	0	0	0.11111	0	0	0	0	0	0
	**152**	0	0	0	0	0.14286	0.125	0	0	0.05	0	0	0	0	0	0	0	0	0	0	0
	**154**	0	0	0	0	0	0	0.025	0.025	0	0	0	0	0	0	0	0	0	0	0	0
	**155**	0	0	0	0	0	0	0	0	0	0	0	0	0	0	0	0	0	0	0.02083	0
	**160**	0	0	0	0	0	0	0	0	0	0	0	0	0	0	0	0	0	0	0.02083	0.02
	**167**	0	0.08333	0	0	0	0	0.025	0.025	0.025	0.03333	0.04167	0	0	0	0	0	0	0.02083	0.04167	0
	**170**	0	0	0	0	0.07143	0	0	0.025	0	0	0	0	0	0	0	0	0	0	0	0
	**172**	0	0.08333	0	0	0	0	0.025	0	0	0.03333	0	0	0	0	0	0	0	0	0	0
	**173**	0	0	0	0	0	0	0	0	0	0	0	0	0	0	0	0	0	0	0.04167	0
	**176**	0	0	0	0	0	0	0	0	0	0	0	0	0	0	0	0	0	0	0	0
	**181**	0	0	0	0	0	0	0	0	0	0	0	0	0	0.05556	0	0	0	0	0	0
	**185**	0	0	0	0	0	0	0	0	0	0	0	0	0.04167	0	0	0	0	0	0	0
	**186**	0	0	0	0	0	0	0	0	0	0	0	0	0	0	0	0	0	0.04167	0	0
	**189**	0.0625	0	0	0	0	0	0	0	0	0	0.02083	0	0	0.02778	0	0.02778	0.02083	0	0.04167	0.08
	**191**	0	0	0	0	0	0	0	0	0	0	0	0	0	0	0	0	0	0.02083	0.02083	0
	**192**	0	0	0	0.25	0	0	0	0	0	0	0	0	0	0	0	0	0	0	0	0
	**193**	0	0	0	0.25	0	0	0	0	0	0	0	0	0	0	0	0	0.02083	0	0.02083	0
	**194**	0	0	0	0	0	0.0625	0	0	0	0	0	0	0	0	0	0	0	0	0	0
	**195**	0	0	0	0	0	0	0	0	0	0	0	0	0	0	0	0	0	0.04167	0	0
	**197**	0	0	0	0	0	0	0.05	0	0	0	0	0	0	0	0	0	0	0	0.02083	0
	**198**	0	0	0	0	0	0	0	0	0	0	0	0	0	0	0	0	0	0	0	0
	**199**	0	0	0	0	0	0	0	0	0	0	0	0	0	0	0	0	0	0	0	0
	**201**	0	0	0	0	0	0	0	0	0	0	0	0	0	0	0.03846	0	0.02083	0	0	0.02
	**202**	0	0	0	0	0.07143	0	0	0.025	0.1	0.06667	0	0	0	0	0	0	0	0	0	0
	**203**	0	0	0.2	0	0	0	0	0	0.025	0	0.125	0	0.02083	0.02778	0.07692	0.05556	0.04167	0.10417	0.0625	0.16
	**204**	0	0	0	0	0	0	0.025	0.025	0	0	0	0	0	0	0	0	0	0	0	0
	**205**	0	0	0	0	0	0	0	0	0	0	0	0	0.02083	0	0.07692	0	0.0625	0	0.04167	0
	**206**	0.0625	0	0	0	0.07143	0	0.025	0.025	0	0.03333	0	0	0	0	0	0	0.02083	0	0	0
	**207**	0	0	0	0	0	0	0	0	0	0	0	0	0.08333	0	0	0	0	0	0	0
	**208**	0	0	0	0	0	0	0.025	0.025	0.05	0.03333	0.02083	0.5	0.02083	0	0	0	0.08333	0.02083	0.02083	0
	**209**	0	0	0	0	0	0	0	0	0	0	0	0	0	0	0	0	0.04167	0	0.02083	0
	**210**	0.1875	0.08333	0.1	0.5	0.07143	0.1875	0.025	0.025	0.1	0.03333	0.04167	0	0.08333	0.22222	0.19231	0	0.10417	0	0.04167	0.04
	**212**	0.25	0.25	0.3	0	0.14286	0.25	0.3	0.325	0.3	0.3	0.39583	0	0.4375	0.30556	0.26923	0.19444	0.375	0.375	0.22917	0.26
	**214**	0.25	0.25	0.1	0	0	0	0.125	0.125	0.05	0.06667	0.08333	0	0.14583	0.02778	0.19231	0.30556	0.08333	0.16667	0.20833	0.24
	**215**	0	0	0	0	0	0	0	0	0	0.06667	0	0	0	0	0	0	0	0	0	0
	**216**	0.1875	0.08333	0	0	0.14286	0	0.075	0.125	0.025	0.03333	0.125	0	0.08333	0	0	0.30556	0.0625	0.10417	0.04167	0.08
	**217**	0	0	0	0	0	0	0	0	0	0	0	0	0	0.11111	0.03846	0	0	0	0	0
	**218**	0	0.08333	0	0	0	0	0.05	0.025	0.05	0.03333	0	0	0	0	0	0	0	0	0	0
	**219**	0	0	0	0	0	0	0	0	0	0	0.04167	0	0	0	0	0	0	0.0625	0	0.04
	**220**	0	0	0	0	0	0	0	0	0	0	0.02083	0	0.0625	0	0.07692	0.02778	0.02083	0.04167	0	0
	**221**	0	0	0	0	0	0	0	0	0	0	0	0	0	0	0	0	0	0	0	0
	**222**	0	0	0	0	0	0	0	0	0	0	0	0	0	0	0	0	0	0	0	0
	**224**	0	0	0.2	0	0	0	0	0	0	0	0	0	0	0	0	0	0	0	0	0
	**225**	0	0	0	0	0	0	0	0	0	0	0	0	0	0	0	0	0.04167	0	0	0

Table S7. Continued.

	Study sites																				
**Locus**	**Allele/n**	**KR 001**	**KR 002**	**KR 003**	**KR 004**	**TW CHU**	**TW JIA**	**TW KAO**	**TW TIC**	**TW TIN**	**TW TIP**	**CN FJS**	**CN SHA**	**CN YUN**	**VN BC1**	**VN BC2**	**VN HCM**	**LA VI1**	**LA VI2**	**LA VI3**	**MM YAN**
**Bi7**	**N**	9	9	5	2	7	8	20	20	20	15	24	0	24	18	13	18	24	24	24	23
	**101**	0	0	0	0	0	0	0	0	0.025	0	0	0	0	0	0	0	0	0	0	0
	**103**	0	0	0	0	0	0	0	0	0	0.06667	0	0	0	0	0	0	0	0	0	0
	**107**	0	0	0	0	0	0	0.05	0	0	0	0	0	0	0	0	0	0	0	0	0
	**108**	0	0	0	0	0	0	0	0	0	0	0.0625	0	0	0	0	0	0	0	0	0
	**109**	0	0	0	0	0	0	0	0	0	0	0.02083	0	0.08333	0	0	0	0	0	0	0
	**115**	0.22222	0.27778	0	0	0	0	0.125	0	0.25	0	0	0	0	0	0	0	0	0	0	0
	**116**	0	0	0	0	0	0	0	0	0.025	0	0	0	0	0	0	0	0	0	0	0
	**117**	0.11111	0.05556	0.1	0	0.07143	0.0625	0.05	0	0.05	0.03333	0	0	0.02083	0.05556	0.03846	0	0	0	0	0
	**118**	0	0	0.3	0	0.07143	0.125	0.025	0.1	0.125	0	0.02083	0	0.125	0	0	0	0	0	0	0
	**119**	0.16667	0	0	0	0	0	0.075	0.05	0.025	0.1	0.04167	0	0.70833	0	0	0	0.08333	0.02083	0.02083	0.08696
	**120**	0	0	0.1	0	0	0	0	0	0	0	0.04167	0	0.02083	0.05556	0	0.08333	0	0	0.04167	0.04348
	**121**	0.05556	0.05556	0	0	0.14286	0.125	0.15	0	0.125	0.13333	0.125	0	0.04167	0	0	0	0.04167	0.02083	0	0
	**122**	0	0	0.1	0.25	0	0	0	0	0	0	0	0	0	0	0	0	0	0.02083	0	0
	**123**	0.05556	0.05556	0.2	0.5	0.07143	0.1875	0	0	0.1	0	0.04167	0	0	0	0	0	0	0	0	0
	**125**	0	0.11111	0	0	0	0.125	0	0.05	0.025	0	0.0625	0	0	0	0	0	0	0	0	0
	**126**	0	0	0	0	0	0	0	0	0	0	0	0	0	0	0	0	0.02083	0	0	0
	**127**	0	0.05556	0	0	0	0.1875	0.075	0.1	0	0	0.0625	0	0	0	0	0	0.125	0.02083	0.04167	0.08696
	**128**	0	0	0	0	0	0	0	0	0	0	0	0	0	0	0	0	0	0	0.04167	0
	**129**	0.05556	0.05556	0.1	0	0.21429	0.125	0.125	0.025	0.05	0.06667	0.16667	0	0	0.05556	0.07692	0.11111	0.14583	0.3125	0.35417	0.23913
	**130**	0	0	0	0	0	0	0	0	0	0	0	0	0	0.05556	0.03846	0	0.02083	0.04167	0.04167	0
	**131**	0.11111	0.11111	0	0	0.28571	0	0.175	0.35	0.025	0.33333	0.20833	0	0	0.30556	0.38462	0.30556	0.35417	0.1875	0.1875	0.13043
	**133**	0.22222	0.22222	0	0	0.07143	0.0625	0.05	0.2	0.1	0.26667	0.14583	0	0	0.30556	0.38462	0.33333	0.14583	0.22917	0.20833	0.32609
	**134**	0	0	0	0	0	0	0	0	0	0	0	0	0	0	0	0	0	0.02083	0	0
	**135**	0	0	0	0	0	0	0	0	0	0	0	0	0	0	0	0	0.0625	0	0	0
	**136**	0	0	0	0	0.07143	0	0	0.075	0.05	0	0	0	0	0	0	0.05556	0	0.02083	0	0
	**138**	0	0	0	0	0	0	0.075	0.05	0.025	0	0	0	0	0.11111	0	0	0	0.08333	0.04167	0
	**140**	0	0	0.1	0.25	0	0	0.025	0	0	0	0	0	0	0.02778	0	0	0	0	0	0
	**141**	0	0	0	0	0	0	0	0	0	0	0	0	0	0	0.07692	0	0	0	0	0
	**142**	0	0	0	0	0	0	0	0	0	0	0	0	0	0.02778	0	0.02778	0	0	0	0
	**143**	0	0	0	0	0	0	0	0	0	0	0	0	0	0	0	0	0	0.02083	0.02083	0
	**144**	0	0	0	0	0	0	0	0	0	0	0	0	0	0	0	0.08333	0	0	0	0.08696
**Locus**	**Allele/n**	**KR 001**	**KR 002**	**KR 003**	**KR 004**	**TW CHU**	**TW JIA**	**TW KAO**	**TW TIC**	**TW TIN**	**TW TIP**	**CN FJS**	**CN SHA**	**CN YUN**	**VN BC1**	**VN BC2**	**VN HCM**	**LA VI1**	**LA VI2**	**LA VI3**	**MM YAN**
**Bt9.1a**	**N**	9	10	5	2	7	8	20	20	20	15	24	1	24	18	12	18	24	24	24	25
	**108**	0	0	0	0	0	0	0	0	0	0	0	0	0	0.02778	0	0	0	0	0	0
	**115**	0	0	0	0	0	0.0625	0.05	0	0.05	0	0	0	0	0	0	0	0	0	0	0
	**117**	0	0	0	0	0	0	0	0.05	0.1	0	0	0	0	0	0	0	0	0	0	0
	**118**	0	0	0	0	0	0	0	0	0	0	0	0	0.02083	0.19444	0.04167	0.05556	0	0	0	0
	**119**	0	0	0	0	0	0	0.025	0.025	0.075	0	0	0	0	0	0	0	0	0	0.04167	0.02
	**120**	0	0	0.3	0.5	0	0	0	0	0	0	0.125	0	0.02083	0	0	0.08333	0.0625	0	0.04167	0.04
	**121**	0	0	0	0	0	0	0.125	0.025	0.1	0.03333	0	0	0.02083	0.02778	0	0	0	0.02083	0	0
	**122**	0	0	0	0	0	0	0	0	0	0	0	0	0	0.02778	0	0	0	0	0	0
	**123**	0.05556	0	0	0	0	0	0	0	0	0	0	0	0.02083	0	0	0.02778	0	0.02083	0	0
	**125**	0	0.05	0	0	0	0.1875	0.025	0.05	0	0	0	0	0.04167	0	0	0	0	0	0	0
	**126**	0	0	0	0	0	0	0	0	0.025	0	0	0	0	0	0	0	0	0.02083	0	0
	**127**	0	0	0	0	0	0.1875	0.075	0.1	0	0.03333	0	0	0.10417	0.08333	0	0	0	0	0	0
	**128**	0	0	0	0	0	0	0	0	0	0	0.02083	0	0	0	0	0.02778	0.04167	0.04167	0.04167	0.08
	**129**	0.33333	0	0.2	0	0.35714	0.125	0.25	0.075	0.05	0.23333	0.375	1	0.22917	0.08333	0.25	0.11111	0.25	0.3125	0.3125	0.18
	**130**	0	0	0	0	0	0	0	0	0	0	0	0	0	0.05556	0.04167	0	0	0.02083	0.02083	0
	**131**	0.11111	0.65	0.3	0.5	0.42857	0	0.25	0.3	0.15	0.43333	0.22917	0	0.27083	0.11111	0.20833	0.41667	0.33333	0.22917	0.27083	0.32
	**133**	0.5	0.3	0.1	0	0.14286	0.125	0.1	0.15	0.225	0.2	0.25	0	0.22917	0.22222	0.20833	0.27778	0.25	0.22917	0.20833	0.36
	**134**	0	0	0	0	0	0	0	0	0	0	0	0	0	0.02778	0	0	0	0	0	0
	**135**	0	0	0	0	0.07143	0	0	0.1	0.075	0.03333	0	0	0	0	0	0	0.0625	0	0	0
	**136**	0	0	0	0	0	0	0	0	0	0	0	0	0.04167	0	0.08333	0	0	0	0	0
	**137**	0	0	0	0	0	0	0	0.025	0	0.03333	0	0	0	0.05556	0	0	0	0	0.04167	0

	**138**	0	0	0	0	0	0	0	0	0	0	0	0	0	0.08333	0.16667	0	0	0.04167	0.02083	0
	**139**	0	0	0	0	0	0.0625	0.075	0.025	0	0	0	0	0	0	0	0	0	0.0625	0	0
	**140**	0	0	0.1	0	0	0	0.025	0.05	0	0	0	0	0	0	0	0	0	0	0	0
	**142**	0	0	0	0	0	0	0	0	0.1	0	0	0	0	0	0	0	0	0	0	0
	**144**	0	0	0	0	0	0.25	0	0	0.025	0	0	0	0	0	0	0	0	0	0	0
	**146**	0	0	0	0	0	0	0	0.025	0	0	0	0	0	0	0	0	0	0	0	0
	**148**	0	0	0	0	0	0	0	0	0	0	0	0	0	0	0	0	0	0	0	0
	**151**	0	0	0	0	0	0	0	0	0.025	0	0	0	0	0	0	0	0	0	0	0

Table S7. Continued.

	**Study sites**																				
**Locus**	**Allele/n**	**KR 001**	**KR 002**	**KR 003**	**KR 004**	**TW CHU**	**TW JIA**	**TW KAO**	**TW TIC**	**TW TIN**	**TW TIP**	**CN FJS**	**CN SHA**	**CN YUN**	**VN BC1**	**VN BC2**	**VN HCM**	**LA VI1**	**LA VI2**	**LA VI3**	**MM YAN**
**Bt7.2b**	**N**	9	10	5	2	7	8	20	20	20	15	24	1	24	18	13	18	24	24	24	25
	**102**	0	0	0	0	0	0	0	0	0	0	0	0	0	0.05556	0.11538	0	0	0	0	0
	**105**	0	0	0	0	0	0	0	0	0	0	0	0	0	0	0.03846	0	0	0	0	0
	**109**	0	0	0	0	0	0	0	0.025	0.05	0	0	0	0	0	0	0	0	0	0	0
	**123**	0	0	0	0	0	0.125	0	0	0	0	0	0	0	0	0	0	0	0	0	0
	**131**	0	0	0	0	0	0	0	0.05	0.025	0	0	0	0	0	0	0	0	0	0	0
	**133**	0	0	0	0	0	0	0	0	0	0	0	0	0	0	0	0	0.02083	0	0	0
	**135**	0	0	0	0	0	0	0.05	0	0	0	0.04167	0	0	0	0	0	0.02083	0	0	0
	**138**	0	0	0	0	0	0	0	0	0.05	0	0	0	0	0	0	0	0	0	0	0
	**139**	0	0	0	0	0	0.125	0	0.075	0	0	0	0	0	0	0	0	0	0	0	0.02
	**140**	0.05556	0	0.2	0	0.21429	0.0625	0.075	0	0.025	0	0	0	0.39583	0.05556	0.07692	0	0	0	0	0
	**141**	0.16667	0.35	0.1	0	0.07143	0.125	0.225	0.45	0.525	0.46667	0	0	0	0	0	0	0.16667	0.1875	0.29167	0
	**142**	0	0.1	0.3	0.5	0	0.0625	0.025	0	0	0	0.54167	0	0.60417	0.5	0.65385	0.38889	0	0.04167	0	0.18
	**143**	0.38889	0.25	0.2	0	0.35714	0.5	0.225	0.35	0.075	0.53333	0.04167	0	0	0	0	0.08333	0.75	0.66667	0.52083	0.06
	**144**	0.33333	0.05	0.1	0.5	0.28571	0	0.3	0	0.125	0	0.375	1	0	0.08333	0.03846	0.52778	0.04167	0.04167	0.125	0.68
	**145**	0.05556	0	0	0	0	0	0	0	0.025	0	0	0	0	0.05556	0	0	0	0	0	0
	**146**	0	0	0	0	0	0	0	0	0	0	0	0	0	0	0	0	0	0	0	0.04
	**147**	0	0	0	0	0	0	0	0.025	0	0	0	0	0	0	0	0	0	0	0.02083	0
	**148**	0	0	0	0	0	0	0	0	0	0	0	0	0	0	0	0	0	0	0	0
	**149**	0	0	0	0	0	0	0	0.025	0	0	0	0	0	0	0	0	0	0	0	0
	**150**	0	0	0	0	0	0	0	0	0	0	0	0	0	0	0	0	0	0	0	0
	**151**	0	0.25	0.1	0	0.07143	0	0.1	0	0.05	0	0	0	0	0.25	0	0	0	0.04167	0	0.02
	**153**	0	0	0	0	0	0	0	0	0	0	0	0	0	0	0	0	0	0.02083	0	0
	**163**	0	0	0	0	0	0	0	0	0	0	0	0	0	0	0	0	0	0	0.02083	0
	**167**	0	0	0	0	0	0	0	0	0	0	0	0	0	0	0	0	0	0	0.02083	0
	**171**	0	0	0	0	0	0	0	0	0	0	0	0	0	0	0.07692	0	0	0	0	0
	**182**	0	0	0	0	0	0	0	0	0	0	0	0	0	0	0	0	0	0	0	0
	**183**	0	0	0	0	0	0	0	0	0.025	0	0	0	0	0	0	0	0	0	0	0
	**187**	0	0	0	0	0	0	0	0	0	0	0	0	0	0	0	0	0	0	0	0
	**189**	0	0	0	0	0	0	0	0	0.025	0	0	0	0	0	0	0	0	0	0	0
**Locus**	**Allele/n**	**KR 001**	**KR 002**	**KR 003**	**KR 004**	**TW CHU**	**TW JIA**	**TW KAO**	**TW TIC**	**TW TIN**	**TW TIP**	**CN FJS**	**CN SHA**	**CN YUN**	**VN BC1**	**VN BC2**	**VN HCM**	**LA VI1**	**LA VI2**	**LA VI3**	**MM YAN**
**Bi11**	**N**	9	10	5	2	7	8	20	20	20	15	24	1	24	18	13	18	24	24	24	25
	**108**	0	0	0	0	0	0	0	0	0	0	0	0	0	0	0	0.13889	0	0	0	0.08
	**110**	0	0	0	0	0	0	0	0	0	0	0	0	0	0.05556	0.15385	0	0	0	0	0
	**120**	0	0	0	0	0	0	0	0	0	0	0	0	0	0	0	0	0	0	0	0
	**123**	0	0	0	0	0	0	0	0	0	0	0	0	0	0.02778	0	0.02778	0	0	0	0
	**126**	0	0	0	0	0	0	0	0	0	0	0	0	0	0	0	0	0	0	0	0
	**127**	0	0	0	0	0	0	0	0	0	0	0	0	0	0	0	0	0	0	0	0.02
	**129**	0	0	0	0	0	0	0	0	0	0	0	0	0	0	0	0	0	0	0	0.04
	**131**	0	0	0	0	0	0	0	0	0	0	0	0	0	0	0	0.02778	0	0	0	0.06
	**133**	0	0	0	0	0	0	0	0	0	0	0	0	0	0	0	0.02778	0	0	0	0.14
	**134**	0	0	0	0	0	0	0	0	0	0	0	0	0	0	0	0	0	0	0	0
	**136**	0	0	0	0	0	0	0	0	0	0	0	0	0	0	0	0	0	0	0	0.04
	**138**	0	0	0	0	0	0	0	0	0	0	0	0	0	0	0	0	0	0	0	0.02
	**140**	0	0	0	0	0	0.0625	0	0	0.025	0	0	0	0	0	0	0	0	0	0	0
	**142**	0	0	0	0	0	0.125	0	0	0	0.03333	0	0	0	0	0	0	0	0	0	0
	**143**	0	0	0	0	0	0	0	0	0	0	0	0	0	0	0	0.41667	0	0	0	0.36

**144**	0	0	0	0	0	0	0	0	0	0	0	0	0	0.02778	0	0	0	0	0	0
**145**	0	0	0	0	0	0	0	0	0	0	0	0	0	0.02778	0	0.22222	0	0	0	0.18
**146**	0	0	0	0	0	0	0	0.025	0	0	0	0	0	0	0	0.02778	0	0	0	0
**148**	0	0.2	0	0	0	0.0625	0	0	0.05	0	0	0	0	0	0	0	0	0	0	0.04
**149**	0	0	0	0	0	0	0	0	0	0	0	0	0	0	0.03846	0	0	0	0	0
**150**	0	0	0	0	0	0	0	0.05	0	0	0	1	0	0	0	0.05556	0	0	0	0.02
**152**	1	0.8	1	1	1	0.6875	1	0.8	0.875	0.9	1	0	1	0.83333	0.80769	0.02778	1	1	1	0
**153**	0	0	0	0	0	0	0	0	0	0	0	0	0	0	0	0	0	0	0	0
**154**	0	0	0	0	0	0	0	0.05	0.05	0.03333	0	0	0	0	0	0	0	0	0	0
**156**	0	0	0	0	0	0	0	0.075	0	0.03333	0	0	0	0	0	0	0	0	0	0
**159**	0	0	0	0	0	0	0	0	0	0	0	0	0	0	0	0.02778	0	0	0	0
**161**	0	0	0	0	0	0	0	0	0	0	0	0	0	0.02778	0	0	0	0	0	0
**166**	0	0	0	0	0	0.0625	0	0	0	0	0	0	0	0	0	0	0	0	0	0
**183**	0	0	0	0	0	0	0	0	0	0	0	0	0	0	0	0	0	0	0	0

Table S7. Continued.

	Study sites																				
**Locus**	**Allele/n**	**KR 001**	**KR 002**	**KR 003**	**KR 004**	**TW CHU**	**TW JIA**	**TW KAO**	**TW TIC**	**TW TIN**	**TW TIP**	**CN FJS**	**CN SHA**	**CN YUN**	**VN BC1**	**VN BC2**	**VN HCM**	**LA VI1**	**LA VI2**	**LA VI3**	**MM YAN**
**Bp181**	**N**	9	10	5	2	7	8	20	20	20	15	24	1	24	18	13	18	24	24	24	25
	**108**	0	0	0	0	0	0	0.075	0.025	0.025	0.03333	0.08333	0	0	0.02778	0	0	0.02083	0	0.02083	0
	**110**	0	0	0	0	0	0	0	0	0	0	0	0	0.0625	0.05556	0	0	0.10417	0.02083	0.14583	0
	**114**	0	0	0	0	0	0	0	0	0	0	0	0	0	0	0	0	0	0	0	0
	**116**	0	0	0	0	0.07143	0	0	0	0.025	0	0	0	0	0	0	0	0	0	0	0
	**117**	0	0	0	0	0	0	0	0	0	0	0	0	0	0	0	0	0	0	0	0
	**118**	0.05556	0.15	0	0	0.07143	0.125	0.2	0.15	0.175	0.23333	0.0625	0	0.08333	0.13889	0.07692	0.02778	0	0.0625	0.0625	0.04
	**119**	0	0	0	0	0	0	0	0	0	0	0	0	0.04167	0.16667	0.53846	0	0	0	0	0
	**120**	0.55556	0.55	0.5	0	0.85714	0.5	0.6	0.65	0.55	0.63333	0.20833	0	0.125	0.16667	0.03846	0.08333	0.125	0.14583	0.1875	0.24
	**121**	0	0	0.4	1	0	0	0	0	0	0	0.27083	0.5	0.20833	0.05556	0.11538	0.63889	0.33333	0.39583	0.20833	0.26
	**122**	0.38889	0.3	0.1	0	0	0.25	0.125	0.175	0.2	0.1	0.375	0	0.47917	0.38889	0.19231	0.19444	0.41667	0.29167	0.3125	0.4
	**123**	0	0	0	0	0	0	0	0	0	0	0	0	0	0	0	0	0	0	0.02083	0.02
	**124**	0	0	0	0	0	0.125	0	0	0	0	0	0	0	0	0.03846	0	0	0	0	0
	**126**	0	0	0	0	0	0	0	0	0.025	0	0	0	0	0	0	0	0	0	0	0
	**127**	0	0	0	0	0	0	0	0	0	0	0	0	0	0	0	0	0	0	0	0
	**130**	0	0	0	0	0	0	0	0	0	0	0	0	0	0	0	0	0	0.02083	0	0
	**132**	0	0	0	0	0	0	0	0	0	0	0	0	0	0	0	0	0	0	0	0
	**136**	0	0	0	0	0	0	0	0	0	0	0	0	0	0	0	0	0	0.02083	0	0.04
	**138**	0	0	0	0	0	0	0	0	0	0	0	0	0	0	0	0	0	0	0.04167	0
	**141**	0	0	0	0	0	0	0	0	0	0	0	0.5	0	0	0	0	0	0.02083	0	0
	**146**	0	0	0	0	0	0	0	0	0	0	0	0	0	0	0	0	0	0	0	0
	**148**	0	0	0	0	0	0	0	0	0	0	0	0	0	0	0	0	0	0.02083	0	0
	**191**	0	0	0	0	0	0	0	0	0	0	0	0	0	0	0	0.05556	0	0	0	0
**Locus**	**Allele/n**	**KR 001**	**KR 002**	**KR 003**	**KR 004**	**TW CHU**	**TW JIA**	**TW KAO**	**TW TIC**	**TW TIN**	**TW TIP**	**CN FJS**	**CN SHA**	**CN YUN**	**VN BC1**	**VN BC2**	**VN HCM**	**LA VI1**	**LA VI2**	**LA VI3**	**MM YAN**
**Bt4.6a**	**N**	9	10	5	2	7	8	20	20	20	15	24	1	24	18	13	18	24	24	24	25
	**114**	0	0	0	0	0	0	0	0	0	0	0	0	0	0	0	0	0	0	0	0
	**116**	0	0	0	0	0	0	0	0	0	0	0	0	0	0	0	0	0.02083	0	0	0
	**118**	0	0	0	0	0	0	0	0	0	0	0	0	0	0.05556	0.03846	0	0	0.02083	0	0.06
	**119**	0	0	0	0	0	0	0	0.025	0	0	0	0	0	0.02778	0	0	0	0	0	0.04
	**120**	0	0	0	0	0	0	0	0	0	0	0	0	0	0.08333	0.19231	0	0	0	0	0
	**121**	0	0	0	0	0	0	0	0	0	0	0	0	0	0	0.03846	0	0	0	0	0
	**122**	0	0	0	0	0	0	0	0	0	0	0	0	0	0.13889	0	0	0.04167	0.02083	0	0
	**125**	0	0	0	0	0	0	0	0	0	0	0	0	0	0	0	0	0	0	0	0
	**127**	0	0.3	0	0	0	0	0	0	0	0	0	0	0	0.02778	0	0	0	0.02083	0	0
	**129**	0.05556	0	0	0	0	0.125	0	0.025	0	0	0.02083	0	0	0	0	0	0	0	0	0.02
	**131**	0	0	0	0	0	0	0	0	0	0	0	0	0	0.02778	0	0	0	0	0	0
	**132**	0	0	0.1	0	0	0	0	0	0	0	0.04167	0	0	0	0	0	0	0	0	0.02
	**133**	0	0	0	0	0.42857	0	0.05	0	0	0	0	0	0	0	0	0	0	0	0	0
	**135**	0.05556	0	0	0.5	0	0	0	0.05	0.025	0	0.02083	0	0	0	0	0	0.02083	0	0	0
	**136**	0	0	0	0	0	0	0	0	0	0	0	0	0.02083	0	0	0	0	0	0	0
	**137**	0.05556	0	0	0	0	0	0.125	0.025	0.075	0	0.0625	0	0.08333	0	0	0	0.08333	0	0	0.08
	**139**	0.05556	0.25	0.1	0	0.21429	0	0.075	0.05	0.05	0.1	0	0	0.125	0.02778	0.07692	0	0.02083	0.08333	0.0625	0.08
	**141**	0.16667	0.25	0.1	0	0	0.125	0.175	0.175	0.175	0.06667	0.04167	1	0.0625	0.13889	0.23077	0.05556	0.04167	0.02083	0	0.06
	**143**	0.11111	0	0	0	0	0.125	0.05	0.15	0.125	0.2	0	0	0	0	0	0	0	0	0	0
	**144**	0	0	0.1	0	0	0	0.025	0	0	0	0.0625	0	0.125	0.11111	0.07692	0.22222	0.08333	0.0625	0.02083	0.12
	**145**	0.16667	0	0	0	0.28571	0.0625	0.2	0.3	0.05	0.23333	0.02083	0	0	0	0	0	0	0	0	0
	**146**	0	0	0.3	0	0	0	0	0	0	0	0.125	0	0.20833	0	0.19231	0.02778	0.16667	0.125	0.02083	0.22
	**147**	0	0	0	0	0	0.0625	0	0	0.1	0.03333	0	0	0	0	0	0.02778	0	0	0	0
	**148**	0.05556	0	0.1	0	0	0.0625	0.075	0.025	0.15	0	0.02083	0	0.08333	0.22222	0.03846	0.02778	0.08333	0.08333	0.04167	0.14
	**149**	0	0	0	0	0	0.0625	0	0.025	0	0	0	0	0	0	0	0.02778	0	0	0	0
	**150**	0	0	0	0	0	0	0.05	0	0.05	0.06667	0	0	0	0	0.07692	0	0	0	0	0
	**151**	0	0	0	0	0	0	0	0	0	0	0.04167	0	0.04167	0.02778	0	0.11111	0	0	0	0.04
	**152**	0	0	0	0	0	0.0625	0	0.025	0.025	0.03333	0	0	0.04167	0	0	0	0	0	0	0
	**153**	0	0	0.2	0	0.07143	0.0625	0.075	0.125	0.05	0.16667	0.0625	0	0	0	0	0	0.04167	0.04167	0.04167	0.04
	**154**	0.27778	0.2	0	0.5	0	0.25	0.1	0	0.025	0	0.27083	0	0	0	0	0.44444	0.375	0.375	0.6875	0.04
	**155**	0	0	0	0	0	0	0	0	0.05	0.03333	0	0	0.125	0.02778	0	0	0	0	0	0
	**156**	0	0	0	0	0	0	0	0	0	0	0.20833	0	0.02083	0.08333	0	0.05556	0	0.02083	0.04167	0.04
	**157**	0	0	0	0	0	0	0	0	0.025	0	0	0	0.02083	0	0.03846	0	0	0	0	0
	**158**	0	0	0	0	0	0	0	0	0	0	0	0	0	0	0	0	0.02083	0	0	0
	**159**	0	0	0	0	0	0	0	0	0	0	0	0	0.02083	0	0	0	0	0	0	0
	**160**	0	0	0	0	0	0	0	0	0	0	0	0	0	0	0	0	0	0.02083	0.04167	0
	**161**	0	0	0	0	0	0	0	0	0	0.03333	0	0	0	0	0	0	0	0.08333	0.04167	0
	**162**	0	0	0	0	0	0	0	0	0	0	0	0	0.02083	0	0	0	0	0	0	0
	**163**	0	0	0	0	0	0	0	0	0.025	0.03333	0	0	0	0	0	0	0	0.02083	0	0

	**164**	0	0	0	0	0	0	0	0	0	0	0	0	0	0	0	0	0	0	0	0
	**165**	0	0	0	0	0	0	0	0	0	0	0	0	0	0	0	0	0	0	0	0

Table S7. Continued.

	**Study sites**																				
**Locus**	**Allele/n**	**KR 001**	**KR 002**	**KR 003**	**KR 004**	**TW CHU**	**TW JIA**	**TW KAO**	**TW TIC**	**TW TIN**	**TW TIP**	**CN FJS**	**CN SHA**	**CN YUN**	**VN BC1**	**VN BC2**	**VN HCM**	**LA VI1**	**LA VI2**	**LA VI3**	**MM YAN**
**Boms60**	**N**	9	10	5	2	7	8	20	20	20	15	24	1	24	18	13	18	24	24	24	25
	**128**	0	0	0	0	0	0	0	0	0	0	0	0	0.02083	0	0	0	0	0	0	0
	**138**	0	0	0	0	0	0	0	0	0	0	0	0	0.02083	0	0	0	0	0	0	0
	**153**	0	0	0	0	0	0	0	0	0	0	0	0	0	0	0	0	0	0	0	0
	**155**	0	0	0	0	0	0.125	0	0	0	0	0	0	0	0	0	0	0	0	0	0
	**156**	0	0	0	0	0	0	0	0	0	0	0	0	0	0	0	0	0	0	0	0
	**161**	0	0	0	0	0	0	0	0	0	0	0	0	0	0	0	0	0	0	0	0
	**163**	0	0	0	0	0	0	0.05	0	0	0	0.02083	0	0	0	0	0	0	0.02083	0	0
	**164**	0	0	0	0	0	0	0	0	0	0.03333	0	0	0	0	0	0	0	0	0.04167	0
	**169**	0	0	0.3	0	0	0	0	0	0	0	0	0	0	0	0	0	0.02083	0	0.04167	0.04
	**170**	0	0	0	0	0	0	0	0	0.025	0	0	0	0.04167	0	0	0	0.04167	0	0.02083	0
	**171**	0	0	0	0	0	0	0.05	0	0	0	0	0	0	0	0	0	0	0	0	0.02
	**173**	0	0	0	0	0	0	0	0	0	0	0	0	0.02083	0	0	0	0	0	0	0
	**174**	0.11111	0	0	0	0	0	0	0	0	0	0	0	0	0	0	0	0	0.02083	0	0
	**176**	0	0	0	0	0	0.0625	0	0	0	0.03333	0	0	0	0	0	0	0	0	0	0
	**177**	0	0	0	0	0	0	0	0	0	0	0	0	0.02083	0.08333	0.03846	0.05556	0	0	0	0
	**178**	0	0	0	0	0	0	0	0	0	0	0	0	0	0	0	0	0	0	0.02083	0
	**179**	0	0	0	0	0	0	0	0	0	0.03333	0	0	0	0	0	0	0	0.0625	0	0.04
	**180**	0.11111	0	0	0	0	0.125	0.125	0.275	0.025	0.06667	0.08333	1	0.04167	0.36111	0.30769	0.47222	0.02083	0.08333	0.08333	0.18
	**181**	0.16667	0	0.1	0	0.42857	0.3125	0.05	0.025	0.225	0.1	0.20833	0	0.0625	0.05556	0.26923	0.11111	0.20833	0.29167	0.125	0.2
	**182**	0.22222	0	0	0	0	0	0.15	0.1	0.075	0.13333	0.04167	0	0.10417	0.13889	0.07692	0.05556	0.10417	0.1875	0.02083	0.12
	**183**	0.11111	0.35	0	0	0.28571	0.0625	0.225	0.275	0.2	0.16667	0.14583	0	0.20833	0.13889	0.03846	0.02778	0.29167	0.14583	0.3125	0.2
	**184**	0.22222	0	0.2	1	0.21429	0.125	0.1	0.05	0.15	0.03333	0.0625	0	0.125	0.02778	0.15385	0	0.08333	0.10417	0.16667	0.08
	**185**	0	0.25	0	0	0	0	0.075	0.025	0	0.16667	0.125	0	0.10417	0.11111	0	0	0.0625	0	0.10417	0.04
	**186**	0	0	0	0	0	0	0.05	0.075	0.025	0.1	0.125	0	0	0	0	0.13889	0.04167	0	0	0.02
	**187**	0	0.05	0.1	0	0	0	0.05	0.05	0.2	0.03333	0.0625	0	0.10417	0.02778	0.03846	0	0.0625	0	0.02083	0.04
	**188**	0.05556	0.35	0	0	0.07143	0.0625	0.025	0.1	0.025	0.06667	0.08333	0	0	0.05556	0.07692	0	0.02083	0.0625	0	0
	**189**	0	0	0.2	0	0	0.0625	0	0	0	0.03333	0	0	0.08333	0	0	0	0.04167	0.02083	0.02083	0
	**190**	0	0	0.1	0	0	0.0625	0.05	0.025	0.05	0	0.04167	0	0.02083	0	0	0	0	0	0.02083	0.02
	**191**	0	0	0	0	0	0	0	0	0	0	0	0	0.02083	0	0	0	0	0	0	0
	**192**	0	0	0	0	0	0	0	0	0	0	0	0	0	0	0	0.13889	0	0	0	0
	**195**	0	0	0	0	0	0	0	0	0	0	0	0	0	0	0	0	0	0	0	0

Table S7. Continued.

	Study sites																				
**Locus**	**Allele/n**	**KR 001**	**KR 002**	**KR 003**	**KR 004**	**TW CHU**	**TW JIA**	**TW KAO**	**TW TIC**	**TW TIN**	**TW TIP**	**CN FJS**	**CN SHA**	**CN YUN**	**VN BC1**	**VN BC2**	**VN HCM**	**LA VI1**	**LA VI2**	**LA VI3**	**MM YAN**
**Bt5**	N	9	7	5	2	7	8	20	20	20	15	24	1	24	18	13	18	24	24	24	22
	**139**	0	0	0	0	0	0	0.025	0	0	0	0	0	0	0	0	0	0	0	0	0
	**146**	0	0	0	0	0	0	0	0	0	0	0	0	0	0	0	0	0	0	0	0
	**148**	0	0	0	0	0	0	0.025	0	0	0	0	0	0	0	0	0	0	0	0	0
	**150**	0	0	0	0	0	0	0	0	0	0	0	0	0	0	0	0	0.02083	0	0	0
	**151**	0	0	0	0	0	0.125	0	0.2	0.25	0.13333	0	0	0	0	0	0	0	0	0	0
	**152**	0	0	0	0	0.07143	0.0625	0.025	0.05	0.025	0.13333	0.02083	0	0	0	0	0	0	0	0	0
	**153**	0	0	0.1	0	0	0	0	0	0	0	0.04167	0	0	0	0	0	0	0	0	0.04545
	**154**	0.11111	0.07143	0	0	0	0	0.025	0	0	0.03333	0	0	0	0	0	0	0	0	0	0
	**155**	0	0	0	0.5	0	0	0	0	0	0	0.08333	0	0	0.02778	0	0.33333	0	0	0	0
	**157**	0	0	0	0	0	0	0	0	0	0.03333	0.14583	0	0	0.02778	0	0	0	0	0	0.04545
	**159**	0	0	0	0	0	0	0	0	0	0.1	0	0	0	0	0	0	0	0	0	0
	**160**	0	0	0	0	0	0	0	0	0	0	0.02083	0	0	0	0	0	0	0	0	0
	**163**	0	0	0	0	0	0	0	0	0.025	0.03333	0.02083	0	0	0	0	0	0	0	0	0
	**166**	0	0	0	0	0	0	0	0	0	0	0	0	0.02083	0	0	0	0	0	0	0
	**168**	0	0	0	0	0	0	0	0	0	0	0	0	0	0	0	0	0	0	0.04167	0
	**169**	0	0	0.1	0	0	0	0	0	0	0	0	0	0	0	0	0	0	0	0	0
	**170**	0	0	0	0	0	0	0.075	0	0	0	0.02083	0	0.08333	0	0	0	0	0	0.02083	0.04545
	**171**	0	0	0	0	0	0	0	0	0	0	0	0	0.02083	0.02778	0	0	0.04167	0	0	0
	**172**	0	0	0.3	0.5	0	0	0	0	0	0	0.33333	0	0.6875	0.08333	0.07692	0.05556	0.4375	0.375	0.375	0.31818
	**173**	0	0	0.2	0	0	0	0.025	0	0	0	0.02083	0	0.125	0.02778	0	0.02778	0.0625	0.0625	0.14583	0.02273
	**175**	0.05556	0.07143	0	0	0	0	0	0.025	0	0.03333	0.08333	0	0.0625	0.05556	0	0	0	0.02083	0	0
	**176**	0	0	0	0	0	0	0	0	0	0	0	0	0	0	0	0	0.08333	0.08333	0	0
	**177**	0	0	0	0	0	0	0	0	0	0	0	0	0	0	0	0	0	0	0	0
	**178**	0	0	0	0	0	0	0	0	0	0	0	0	0	0.05556	0	0.05556	0	0	0	0
	**179**	0.16667	0.07143	0	0	0	0.0625	0	0.05	0.025	0	0	0	0	0	0	0	0	0	0.0625	0.02273
	**180**	0	0	0	0	0	0	0	0	0	0	0	0	0	0.11111	0.30769	0.13889	0.0625	0.125	0.0625	0
	**181**	0.22222	0	0.1	0	0.42857	0.375	0.275	0.3	0.25	0.13333	0.0625	1	0	0.27778	0.26923	0.13889	0.125	0.10417	0.10417	0.15909
	**182**	0	0	0	0	0	0	0	0	0	0	0	0	0	0.11111	0.15385	0	0	0	0	0
	**183**	0.33333	0.35714	0	0	0.42857	0.25	0.35	0.15	0.075	0.26667	0.04167	0	0	0.08333	0.03846	0.02778	0.08333	0.1875	0.10417	0.11364
	**184**	0	0	0	0	0	0	0	0	0	0	0	0	0	0	0.07692	0	0	0	0	0
	**185**	0.11111	0.14286	0	0	0	0	0.075	0.1	0.2	0.06667	0.10417	0	0	0.02778	0.07692	0	0.04167	0.02083	0.04167	0.11364
	**186**	0	0	0	0	0	0	0	0	0	0	0	0	0	0	0	0.08333	0	0.02083	0.04167	0.06818
	**187**	0	0.28571	0.1	0	0.07143	0.125	0.05	0.075	0.1	0.03333	0	0	0	0.02778	0	0.08333	0.02083	0	0	0.02273
	**188**	0	0	0	0	0	0	0	0	0	0	0	0	0	0.05556	0	0	0	0	0	0
	**189**	0	0	0.1	0	0	0	0.025	0.05	0.05	0	0	0	0	0	0	0	0	0	0	0.02273
	**191**	0	0	0	0	0	0	0.025	0	0	0	0	0	0	0	0	0.02778	0.02083	0	0	0
	**195**	0	0	0	0	0	0	0	0	0	0	0	0	0	0	0	0.02778	0	0	0	0
	**199**	0	0	0	0	0	0	0	0	0	0	0	0	0	0	0	0	0	0	0	0

Table S7. Continued.

	Study sites																				
**Locus**	**Allele/n**	**TH BAN**	**TH CHA**	**TH DA1**	**TH DA2**	**KH PNP**	**IN DEL**	**NP POK**	**PH BUS**	**PH GUG**	**PH GUZ**	**PH LU1**	**PH LU2**	**PH LU3**	**PH MIN**	**PH PA1**	**PH PA2**	**PH PA3**	**PH PA4**	**PH PA5**	**MY PEN**
**Bp200**	**N**	5	4	20	16	1	9	1	4	24	8	6	13	28	13	6	22	8	9	33	24
	**94**	0	0	0	0	0	0	0	0	0	0	0	0	0	0	0	0	0	0	0	0
	**98**	0	0	0	0	0	0	0	0	0.02083	0.0625	0	0	0.01786	0.03846	0	0	0.0625	0.05556	0	0
	**100**	0	0.125	0	0.09375	0	0	0	0	0.04167	0	0	0	0.07143	0	0	0.06818	0	0	0.09091	0
	**101**	0	0	0	0	0	0	0	0	0	0	0	0	0	0	0	0	0	0	0	0
	**102**	0	0	0.15	0.125	0	0	0	0.125	0	0	0	0	0.03571	0	0	0	0	0.05556	0	0.04167
	**103**	0	0	0	0	0	0	0	0	0	0	0	0	0	0	0	0	0	0	0	0
	**104**	0.7	0.25	0.375	0.40625	0	0.22222	0	0.25	0.27083	0.4375	0.58333	0.5	0.35714	0.65385	0.33333	0.31818	0.4375	0.5	0.36364	0.25
	**106**	0.2	0.375	0.4	0.25	1	0.66667	0	0.5	0.04167	0.3125	0.25	0.34615	0.16071	0.11538	0.33333	0.34091	0.375	0.16667	0.33333	0.70833
	**107**	0	0	0	0	0	0	0	0	0	0	0	0	0	0	0	0	0	0	0	0
	**108**	0	0.125	0.025	0.0625	0	0	0	0.125	0.125	0	0	0.03846	0.125	0.03846	0.16667	0.11364	0.0625	0.05556	0.12121	0
	**109**	0	0	0	0	0	0	0	0	0	0	0	0	0	0	0	0	0	0	0	0
	**110**	0	0	0.025	0	0	0	0	0	0.45833	0.125	0	0.03846	0.17857	0	0.16667	0.13636	0	0.11111	0.09091	0
	**118**	0	0	0	0	0	0	1	0	0.02083	0.0625	0	0	0.01786	0	0	0	0	0	0	0
	**120**	0	0.125	0	0	0	0	0	0	0	0	0	0	0	0	0	0	0	0	0	0
	**122**	0	0	0	0.0625	0	0	0	0	0	0	0	0	0	0	0	0	0	0	0	0
	**124**	0.1	0	0	0	0	0	0	0	0	0	0	0	0	0	0	0	0	0	0	0
	**126**	0	0	0.025	0	0	0.11111	0	0	0.02083	0	0.16667	0.07692	0.03571	0.15385	0	0.02273	0.0625	0.05556	0	0
	**128**	0	0	0	0	0	0	0	0	0	0	0	0	0	0	0	0	0	0	0	0
**Locus**	**Allele/n**	**TH BAN**	**TH CHA**	**TH DA1**	**TH DA2**	**KH PNP**	**IN DEL**	**NP POK**	**PH BUS**	**PH GUG**	**PH GUZ**	**PH LU1**	**PH LU2**	**PH LU3**	**PH MIN**	**PH PA1**	**PH PA2**	**PH PA3**	**PH PA4**	**PH PA5**	**MY PEN**
**Bi4**	**N**	5	4	20	16	1	9	1	4	24	8	6	13	28	13	6	22	8	9	33	24
	**111**	0	0	0	0	0	0	0	0	0	0	0	0	0	0	0	0	0	0	0	0
	**112**	0	0	0	0	0	0	0	0	0	0	0	0	0	0	0	0	0	0	0	0
	**114**	0	0	0	0	0	0	0	0	0	0	0	0	0	0	0	0.02273	0	0	0	0
	**115**	0	0	0	0	0	0	0	0	0	0	0	0	0	0	0	0	0	0	0	0
	**120**	0	0.25	0.05	0	0	0	0	0	0	0	0	0	0	0	0	0	0	0	0	0
	**122**	0	0	0	0	0	0	0	0	0	0	0	0	0	0	0	0	0	0	0	0
	**123**	0	0	0.05	0.34375	0	0	0	0	0	0	0	0	0	0	0	0	0	0	0	0
	**124**	0	0.125	0	0	0	0	0	0	0	0	0	0	0	0	0	0	0	0	0	0
	**125**	0.2	0	0.075	0	0	0	0	0.25	0.0625	0.125	0	0.15385	0.07143	0.03846	0.25	0.20455	0.0625	0.11111	0.24242	0
	**126**	0.6	0.375	0.6	0.625	1	0.77778	1	0.625	0.75	0.8125	1	0.69231	0.80357	0.84615	0.66667	0.54545	0.875	0.61111	0.56061	0.85417
	**127**	0	0	0	0	0	0	0	0	0	0	0	0	0	0	0	0.02273	0	0	0.0303	0
	**128**	0.2	0.125	0.15	0.03125	0	0.22222	0	0.125	0.16667	0.0625	0	0.15385	0.125	0.11538	0.08333	0.20455	0.0625	0.27778	0.16667	0.14583
	**129**	0	0	0	0	0	0	0	0	0	0	0	0	0	0	0	0	0	0	0	0
	**130**	0	0	0.025	0	0	0	0	0	0.02083	0	0	0	0	0	0	0	0	0	0	0
	**133**	0	0	0.05	0	0	0	0	0	0	0	0	0	0	0	0	0	0	0	0	0
	**134**	0	0	0	0	0	0	0	0	0	0	0	0	0	0	0	0	0	0	0	0
	**136**	0	0.125	0	0	0	0	0	0	0	0	0	0	0	0	0	0	0	0	0	0
	**139**	0	0	0	0	0	0	0	0	0	0	0	0	0	0	0	0	0	0	0	0

Table S7. Continued.

	Study sites																				
**Locus**	**Allele/n**	**TH BAN**	**TH CHA**	**TH DA1**	**TH DA2**	**KH PNP**	**IN DEL**	**NP POK**	**PH BUS**	**PH GUG**	**PH GUZ**	**PH LU1**	**PH LU2**	**PH LU3**	**PH MIN**	**PH PA1**	**PH PA2**	**PH PA3**	**PH PA4**	**PH PA5**	**MY PEN**
**Bt5.8a**	N	5	4	20	16	1	9	1	4	24	8	6	13	28	13	6	22	8	9	33	24
	**101**	0	0	0	0.125	0	0	0	0	0	0	0	0	0	0	0	0	0	0	0	0
	**105**	0	0.125	0	0	0	0	0	0	0	0	0	0	0	0	0	0	0	0	0	0
	**106**	0	0	0	0	0	0	0	0	0	0	0	0	0	0	0	0	0	0	0	0
	**107**	0	0	0	0.03125	0	0	0	0	0	0	0	0	0	0	0	0	0	0	0	0
	**109**	0	0	0	0	0	0	0	0	0	0	0	0	0	0	0	0	0	0	0	0
	**111**	0	0.125	0	0	0	0	0	0	0	0	0	0	0	0	0	0	0	0	0	0
	**112**	0	0	0	0	0	0	0	0	0	0	0	0	0	0	0	0	0	0	0	0
	**113**	0	0	0.05	0	0	0	0	0	0	0	0	0.07692	0.01786	0.11538	0	0	0	0	0	0
	**114**	0	0	0	0	0	0	0	0	0	0	0	0	0	0	0	0.02273	0	0	0	0
	**115**	0	0	0	0.0625	0	0	0	0	0	0	0	0	0.03571	0.11538	0	0	0.0625	0	0	0.02083
	**116**	0	0	0	0	0	0	0	0	0	0	0	0	0	0	0	0	0	0	0	0
	**117**	0	0	0	0	0	0	0	0	0	0	0	0	0	0	0	0	0	0	0	0
	**118**	0	0	0	0	0	0	0	0	0	0	0	0	0	0	0	0	0	0	0	0
	**119**	0	0	0.05	0	0	0	0	0	0	0	0	0	0	0	0	0	0	0	0	0
	**120**	0	0	0	0	0	0	0	0	0	0	0	0	0	0	0	0	0	0	0	0
	**121**	0.2	0.625	0.1	0.1875	0	0	0	0	0.0625	0.0625	0	0.03846	0.23214	0	0.08333	0	0	0	0	0
	**122**	0	0	0	0	0	0.11111	0	0.125	0.29167	0	0	0.03846	0.03571	0	0.16667	0.13636	0	0.16667	0.15152	0.33333
	**123**	0	0	0	0.0625	0	0	0	0	0	0	0	0	0	0	0.08333	0	0	0	0	0
	**124**	0	0	0.15	0.1875	0	0	0	0	0.02083	0	0	0	0	0	0	0	0	0	0	0.02083
	**125**	0.4	0	0	0.0625	0	0	0	0	0	0	0	0	0	0	0	0	0	0	0	0
	**126**	0	0	0	0	0	0	0	0	0	0	0	0	0	0	0	0	0	0	0	0
	**127**	0	0	0	0	0	0	0	0	0	0	0	0	0	0	0	0	0	0	0	0
	**128**	0.3	0.125	0.475	0.21875	0	0.33333	0.5	0.5	0.20833	0.5	0.5	0.38462	0.41071	0.53846	0.16667	0.22727	0.375	0.27778	0.40909	0.4375
	**130**	0.1	0	0.175	0.03125	0	0	0	0.125	0.02083	0.125	0.08333	0.15385	0.10714	0.03846	0.16667	0.15909	0.0625	0.05556	0.07576	0.1875
	**132**	0	0	0	0	0	0	0	0	0	0	0	0	0	0	0	0	0	0	0	0
	**133**	0	0	0	0	0	0	0	0	0	0	0	0	0	0	0	0	0	0	0	0
	**134**	0	0	0	0	0	0	0	0	0	0	0	0	0	0	0	0	0	0	0	0
	**137**	0	0	0	0.03125	0	0	0	0	0	0	0	0	0	0	0	0	0	0	0	0
	**138**	0	0	0	0	0	0	0	0	0	0	0	0	0	0	0	0	0	0	0	0
	**139**	0	0	0	0	0	0	0	0	0	0	0	0	0	0	0	0	0	0	0	0
	**144**	0	0	0	0	0	0	0	0	0	0	0	0	0	0	0	0	0	0	0.01515	0
	**150**	0	0	0	0	0	0	0	0	0	0	0	0	0	0	0	0	0	0	0	0
	**151**	0	0	0	0	0	0	0	0	0	0	0	0	0	0	0	0	0	0	0	0
	**152**	0	0	0	0	1	0.55556	0.5	0.25	0.39583	0.3125	0.41667	0.30769	0.16071	0.19231	0.33333	0.45455	0.5	0.5	0.34848	0
	**157**	0	0	0	0	0	0	0	0	0	0	0	0	0	0	0	0	0	0	0	0
	**160**	0	0	0	0	0	0	0	0	0	0	0	0	0	0	0	0	0	0	0	0

Table S7. Continued.

	Study sites																				
**Locus**	**Allele/n**	**TH BAN**	**TH CHA**	**TH DA1**	**TH DA2**	**KH PNP**	**IN DEL**	**NP POK**	**PH BUS**	**PH GUG**	**PH GUZ**	**PH LU1**	**PH LU2**	**PH LU3**	**PH MIN**	**PH PA1**	**PH PA2**	**PH PA3**	**PH PA4**	**PH PA5**	**MY PEN**
**Bt4**	N	5	4	20	16	1	9	1	4	24	8	6	13	28	13	6	22	8	9	33	24
	**100**	0	0	0	0	0	0	0	0	0	0	0	0	0	0	0	0	0	0	0	0
	**102**	0	0	0	0	0	0	0	0	0	0	0	0	0	0	0	0	0	0	0	0
	**103**	0	0	0	0	0	0	0	0	0.02083	0	0	0	0	0	0	0.04545	0	0	0	0
	**104**	0	0	0	0	0	0	0	0	0	0	0	0	0	0	0	0	0	0	0	0
	**107**	0	0	0	0	0	0	0	0	0	0	0	0	0	0	0	0	0	0	0	0
	**113**	0	0	0	0	0	0	0	0	0	0	0	0	0	0	0	0	0	0	0	0
	**115**	0	0	0	0	0	0	0	0	0	0	0	0	0.01786	0	0	0	0	0	0.01515	0.02083
	**116**	0	0	0	0	0	0	0	0	0	0.0625	0	0	0	0	0	0	0	0	0	0
	**117**	0.2	0	0.05	0	0	0	0	0	0	0	0	0	0	0	0	0	0	0	0	0
	**118**	0	0	0.025	0	0	0	0	0	0	0.0625	0	0	0	0	0	0	0	0	0	0
	**119**	0	0	0	0	0	0	0	0	0	0	0	0	0	0	0	0	0	0	0	0
	**120**	0.2	0.25	0	0.03125	1	0.11111	0	0	0	0.0625	0	0	0	0.03846	0	0	0	0	0.01515	0.1875
	**121**	0	0	0	0	0	0	0	0	0	0	0	0	0	0	0	0	0	0	0	0
	**122**	0.2	0.25	0.075	0.03125	0	0.16667	0	0	0	0.25	0.16667	0.03846	0.07143	0.19231	0.08333	0.09091	0.125	0.11111	0.13636	0.20833
	**124**	0.2	0	0.25	0.1875	0	0.05556	0	0.5	0.58333	0.125	0.33333	0.34615	0.41071	0.15385	0.41667	0.43182	0	0.61111	0.48485	0.29167
	**126**	0	0	0.075	0.21875	0	0.33333	0	0.25	0.125	0.25	0.25	0.11538	0.14286	0.15385	0.25	0.25	0.375	0.11111	0.24242	0.1875
	**128**	0	0	0.225	0.15625	0	0.16667	0	0.25	0.14583	0.0625	0.25	0.26923	0.26786	0.15385	0.25	0.11364	0.3125	0.05556	0.07576	0.02083
	**130**	0	0.25	0.1	0	0	0.05556	1	0	0.04167	0	0	0.03846	0.03571	0.11538	0	0.02273	0.0625	0.05556	0	0.08333
	**132**	0.2	0	0.125	0.09375	0	0.05556	0	0	0.02083	0	0	0.03846	0.01786	0.03846	0	0	0.0625	0	0.01515	0
	**133**	0	0	0	0	0	0	0	0	0	0	0	0	0	0	0	0	0	0	0	0
	**134**	0	0	0	0.21875	0	0	0	0	0.02083	0.0625	0	0.11538	0.03571	0.11538	0	0	0.0625	0.05556	0	0
	**135**	0	0	0	0	0	0	0	0	0	0.0625	0	0	0	0	0	0	0	0	0	0
	**136**	0	0	0.025	0	0	0	0	0	0.04167	0	0	0.03846	0	0	0	0	0	0	0	0
	**137**	0	0	0	0	0	0	0	0	0	0	0	0	0	0	0	0	0	0	0	0
	**139**	0	0	0	0	0	0.05556	0	0	0	0	0	0	0	0	0	0	0	0	0.01515	0
	**140**	0	0.25	0.05	0	0	0	0	0	0	0	0	0	0	0.03846	0	0	0	0	0	0
	**141**	0	0	0	0	0	0	0	0	0	0	0	0	0	0	0	0	0	0	0	0
	**146**	0	0	0	0.0625	0	0	0	0	0	0	0	0	0	0	0	0.04545	0	0	0	0
	**148**	0	0	0	0	0	0	0	0	0	0	0	0	0	0	0	0	0	0	0	0
	**156**	0	0	0	0	0	0	0	0	0	0	0	0	0	0	0	0	0	0	0	0

Table S7. Continued.

	Study sites																				
**Locus**	**Allele/n**	**TH BAN**	**TH CHA**	**TH DA1**	**TH DA2**	**KH PNP**	**IN DEL**	**NP POK**	**PH BUS**	**PH GUG**	**PH GUZ**	**PH LU1**	**PH LU2**	**PH LU3**	**PH MIN**	**PH PA1**	**PH PA2**	**PH PA3**	**PH PA4**	**PH PA5**	**MY PEN**
**Bi8**	**N**	5	4	20	16	1	9	1	4	22	6	6	12	28	13	5	22	8	8	33	24
	**99**	0	0	0	0	0	0	0	0	0	0	0	0	0	0	0	0	0	0	0	0
	**104**	0	0	0	0	0	0	0	0	0	0	0	0	0	0	0	0	0	0	0	0
	**110**	0	0	0	0	0	0	0	0	0	0	0	0	0	0	0	0	0	0	0	0
	**113**	0	0	0	0	0	0	0	0	0	0	0	0	0.03571	0	0	0	0	0	0	0
	**114**	0	0	0	0	0	0	0	0	0	0	0	0	0	0	0	0	0.0625	0	0	0
	**117**	0	0	0.025	0	0	0	0	0	0	0	0	0	0.01786	0	0	0	0	0	0	0
	**120**	0	0	0.05	0.0625	0	0.11111	0	0	0.02273	0.08333	0	0.04167	0.10714	0.11538	0.1	0.11364	0	0.0625	0.15152	0.02083
	**121**	0.2	0.25	0	0	0	0	0	0	0	0	0	0.08333	0	0	0	0	0	0	0	0
	**123**	0	0	0.05	0.09375	0	0	0	0.125	0	0	0	0	0	0	0	0	0	0	0	0
	**124**	0	0	0	0	0	0	0	0	0	0	0	0	0	0	0	0	0	0	0	0
	**125**	0	0	0	0	0	0	0	0	0	0	0	0	0	0	0	0	0.0625	0	0.01515	0
	**127**	0	0	0	0	0	0	0	0	0	0	0	0	0	0	0	0	0	0	0	0
	**129**	0	0	0.025	0	0	0	0	0	0	0	0	0	0	0	0	0	0	0	0	0
	**130**	0	0	0	0	0	0	0	0	0	0	0	0	0	0	0	0	0	0	0	0
	**131**	0	0.25	0.1	0	0	0.05556	0	0	0	0	0.08333	0	0.01786	0	0	0	0	0	0	0.20833
	**132**	0	0	0	0	0	0	0	0	0	0	0	0	0	0	0	0	0	0	0	0
	**133**	0	0	0	0	0	0.05556	0	0	0	0	0	0.04167	0.01786	0	0	0.02273	0	0	0	0.02083
	**135**	0.4	0.25	0.225	0.28125	0	0.38889	0	0.625	0.75	0.75	0.91667	0.75	0.42857	0.65385	0.7	0.75	0.6875	0.6875	0.62121	0.375
	**136**	0	0	0	0	0	0	0	0	0	0	0	0	0	0	0	0	0	0	0	0
	**137**	0	0	0.175	0.3125	0	0	0	0.125	0.13636	0.08333	0	0	0.14286	0	0	0.02273	0.125	0.125	0.04545	0
	**138**	0	0	0	0	0	0	0	0	0	0	0	0	0	0	0	0	0	0	0	0
	**139**	0.4	0	0.15	0.25	1	0.38889	1	0.125	0.09091	0.08333	0	0	0.14286	0.15385	0.2	0.09091	0.0625	0.125	0.16667	0.35417
	**140**	0	0	0	0	0	0	0	0	0	0	0	0	0	0	0	0	0	0	0	0
	**141**	0	0	0.05	0	0	0	0	0	0	0	0	0	0	0	0	0	0	0	0	0.02083
	**143**	0	0	0.1	0	0	0	0	0	0	0	0	0	0	0	0	0	0	0	0	0
	**144**	0	0	0	0	0	0	0	0	0	0	0	0	0.03571	0	0	0	0	0	0	0
	**145**	0	0	0.025	0	0	0	0	0	0	0	0	0	0.01786	0	0	0	0	0	0	0
	**146**	0	0.25	0	0	0	0	0	0	0	0	0	0.08333	0	0.07692	0	0	0	0	0	0
	**147**	0	0	0.025	0	0	0	0	0	0	0	0	0	0.03571	0	0	0	0	0	0	0
	**149**	0	0	0	0	0	0	0	0	0	0	0	0	0	0	0	0	0	0	0	0

Table S7. Continued.

	Study sites																				
**Locus**	**Allele/n**	**TH BAN**	**TH CHA**	**TH DA1**	**TH DA2**	**KH PNP**	**IN DEL**	**NP POK**	**PH BUS**	**PH GUG**	**PH GUZ**	**PH LU1**	**PH LU2**	**PH LU3**	**PH MIN**	**PH PA1**	**PH PA2**	**PH PA3**	**PH PA4**	**PH PA5**	**MY PEN**
**Bt6.8a**	**N**	5	4	20	16	1	9	1	4	24	8	6	13	28	13	6	22	8	9	33	24
	**103**	0	0	0	0	0	0	0	0	0	0	0	0	0	0	0	0	0	0	0	0
	**104**	0	0	0	0	0	0	0	0	0	0	0	0	0	0	0	0	0	0	0	0
	**109**	0	0	0	0	0	0	0	0	0	0	0	0	0	0	0	0	0	0	0	0
	**111**	0	0	0	0	0	0	0	0	0	0	0	0	0	0	0	0	0	0	0	0
	**117**	0	0	0	0	0	0	0	0	0	0	0	0	0	0	0	0	0	0	0	0
	**118**	0	0	0	0	0	0	0	0	0	0	0	0	0	0	0	0	0	0	0	0
	**119**	0	0	0.05	0	0	0	0	0	0	0	0	0	0	0	0	0	0	0	0	0
	**132**	0	0	0	0.0625	0	0	0	0	0	0	0	0	0	0	0	0	0	0	0	0
	**133**	0	0.25	0	0	0	0	0	0	0	0	0	0	0	0	0	0	0	0	0	0
	**135**	0	0	0	0	0	0.05556	0	0	0	0	0	0	0	0	0.08333	0	0	0	0	0
	**137**	0	0	0	0	0	0	0	0	0	0	0	0	0	0	0	0	0	0	0	0.02083
	**140**	0	0	0	0	0	0	0	0	0.02083	0	0	0.03846	0.01786	0.03846	0	0	0.125	0.05556	0.01515	0
	**141**	0	0.375	0	0	0	0	0	0	0	0	0	0.07692	0.08929	0.07692	0	0	0	0	0	0
	**142**	0	0	0	0	0	0	0	0	0.02083	0.0625	0	0	0	0	0	0.02273	0	0.05556	0	0
	**144**	0	0	0.125	0.34375	0	0.22222	0	0	0.02083	0.1875	0	0	0.03571	0	0	0	0.125	0	0.01515	0
	**146**	0	0	0	0	0	0	0	0	0.54167	0	0	0.19231	0.03571	0.03846	0.16667	0.25	0.0625	0.22222	0.18182	0
	**147**	0	0	0	0	0	0	0	0.125	0.0625	0.0625	0	0.11538	0.26786	0.15385	0.08333	0	0	0.05556	0	0.02083
	**150**	0.3	0	0.025	0.125	0	0	0	0.125	0.1875	0.125	0	0.15385	0.14286	0.23077	0.08333	0.13636	0.1875	0.05556	0.07576	0.0625
	**151**	0	0	0	0	0	0.16667	0.5	0	0	0	0	0	0	0	0	0	0	0	0	0
	**153**	0.3	0	0.05	0	0	0	0	0	0	0.0625	0.16667	0	0.03571	0	0	0	0	0	0	0
	**154**	0	0	0	0	0.5	0.27778	0	0.375	0.0625	0.25	0.5	0.30769	0.19643	0.11538	0.16667	0.22727	0.125	0.33333	0.40909	0.125
	**156**	0	0	0	0	0	0	0	0	0	0	0	0	0	0	0	0	0	0	0	0
	**157**	0	0	0	0	0	0	0	0	0	0	0	0	0	0	0	0	0	0	0	0
	**159**	0.4	0.375	0.4	0.46875	0.5	0.11111	0	0.375	0.04167	0.25	0.33333	0.11538	0.07143	0.34615	0.33333	0.29545	0.375	0.22222	0.24242	0.5625
	**161**	0	0	0	0	0	0	0	0	0	0	0	0	0	0	0	0	0	0	0	0
	**162**	0	0	0.05	0	0	0	0	0	0	0	0	0	0	0	0	0	0	0	0	0
	**168**	0	0	0.1	0	0	0	0	0	0.02083	0	0	0	0.01786	0	0	0	0	0	0	0
	**169**	0	0	0	0	0	0.16667	0.5	0	0.02083	0	0	0	0.03571	0	0	0.04545	0	0	0.01515	0.14583
	**171**	0	0	0.025	0	0	0	0	0	0	0	0	0	0	0	0	0	0	0	0	0
	**173**	0	0	0	0	0	0	0	0	0	0	0	0	0	0	0	0	0	0	0	0
	**174**	0	0	0.175	0	0	0	0	0	0	0	0	0	0.01786	0	0	0	0	0	0	0.04167
	**175**	0	0	0	0	0	0	0	0	0	0	0	0	0.01786	0	0.08333	0.02273	0	0	0.04545	0
	**178**	0	0	0	0	0	0	0	0	0	0	0	0	0	0	0	0	0	0	0	0
	**181**	0	0	0	0	0	0	0	0	0	0	0	0	0	0	0	0	0	0	0	0
	**182**	0	0	0	0	0	0	0	0	0	0	0	0	0	0	0	0	0	0	0	0
	**184**	0	0	0	0	0	0	0	0	0	0	0	0	0	0	0	0	0	0	0	0
	**186**	0	0	0	0	0	0	0	0	0	0	0	0	0	0	0	0	0	0	0	0
	**189**	0	0	0	0	0	0	0	0	0	0	0	0	0	0	0	0	0	0	0	0.02083
	**190**	0	0	0	0	0	0	0	0	0	0	0	0	0.01786	0	0	0	0	0	0	0

Table S7. Continued.

	Study sites																				
**Locus**	**Allele/n**	**TH BAN**	**TH CHA**	**TH DA1**	**TH DA2**	**KH PNP**	**IN DEL**	**NP POK**	**PH BUS**	**PH GUG**	**PH GUZ**	**PH LU1**	**PH LU2**	**PH LU3**	**PH MIN**	**PH PA1**	**PH PA2**	**PH PA3**	**PH PA4**	**PH PA5**	**MY PEN**
**Boms3a**	**N**	5	4	20	16	1	9	1	4	21	7	6	11	27	12	5	22	8	9	33	24
	**102**	0	0	0.05	0.21875	0	0	0	0	0	0	0	0	0.09259	0	0	0	0	0	0	0
	**106**	0	0	0	0	0	0	0	0	0	0	0	0	0	0	0	0	0	0	0	0
	**108**	0	0	0	0	0	0.11111	0	0	0.04762	0	0	0	0	0	0	0.09091	0	0	0	0
	**110**	0	0	0	0	0	0	0	0	0	0	0	0	0	0.08333	0	0	0	0	0	0
	**115**	0	0	0	0	0	0	0	0	0	0	0	0	0	0	0	0	0	0	0	0
	**117**	0	0	0	0	0	0	0	0	0	0	0	0	0	0	0	0	0	0	0	0
	**120**	0.1	0	0	0.03125	0	0	0	0	0.02381	0	0	0	0	0	0	0	0	0	0	0
	**124**	0	0	0	0	0	0	0	0	0.02381	0	0	0	0	0	0	0	0	0	0.01515	0
	**126**	0	0	0	0	0	0	0	0	0	0	0	0	0	0	0	0	0	0	0	0
	**133**	0	0	0	0.125	0	0	0	0.25	0	0	0	0	0	0	0	0	0	0	0	0
	**134**	0	0	0	0	0	0	0	0	0	0	0	0	0	0	0	0	0	0	0	0
	**135**	0	0	0	0	0	0	0	0	0.14286	0.14286	0	0.04545	0.01852	0.08333	0.2	0.02273	0	0	0.07576	0.02083
	**136**	0	0	0	0.0625	0	0	0	0	0	0	0	0	0	0	0	0	0	0.05556	0	0
	**138**	0	0	0	0	0	0	0	0	0	0	0	0	0.01852	0	0	0	0	0	0	0
	**139**	0	0	0	0	0	0	0	0	0	0	0	0	0	0	0	0	0	0	0	0.04167
	**140**	0	0	0	0.0625	0	0	0	0	0	0	0	0	0.01852	0	0	0	0	0.05556	0	0
	**142**	0	0	0	0	0	0	0	0	0	0	0	0	0	0	0	0	0	0	0	0
	**144**	0	0	0	0	0	0	0	0	0	0	0	0	0	0	0	0.02273	0	0	0	0
	**147**	0	0	0	0	0	0	0	0	0.02381	0	0	0.09091	0	0	0	0	0	0	0	0
	**150**	0	0	0	0	0	0	0	0	0	0	0	0	0	0	0	0	0	0	0	0
	**152**	0	0	0	0	0	0	0	0	0	0	0	0	0.03704	0	0	0	0	0	0	0
	**154**	0	0	0	0	0	0	0	0	0	0	0	0	0	0	0	0	0	0	0	0
	**155**	0	0	0	0	0	0	0	0	0	0	0	0	0	0	0	0	0	0	0	0
	**160**	0	0	0	0	0	0	0	0	0.02381	0	0	0.09091	0	0.04167	0	0	0	0	0	0
	**167**	0	0	0.025	0	0	0	0	0	0	0	0	0	0	0	0	0	0	0	0	0.04167
	**170**	0	0	0	0	0	0	0	0	0	0	0	0	0	0	0	0	0	0	0	0
	**172**	0	0	0.025	0	0	0	0	0	0	0	0	0	0	0	0	0	0	0	0	0
	**173**	0	0	0	0	0	0	0	0	0	0	0	0	0	0	0	0	0	0	0	0
	**176**	0.2	0.5	0	0	0	0	0	0	0	0	0	0.09091	0	0	0	0	0	0	0	0
	**181**	0	0	0	0	0	0	0	0	0	0	0	0	0	0	0	0	0	0	0	0
	**185**	0	0	0	0	0	0	0	0	0	0	0	0	0	0	0	0	0	0	0	0
	**186**	0	0	0	0	0	0	0	0	0	0	0	0	0.01852	0	0	0	0	0	0	0
	**189**	0	0	0.025	0	0	0	0	0	0	0	0	0	0	0	0	0	0	0	0.01515	0.04167
	**191**	0	0	0	0	0	0.05556	0	0	0.02381	0	0	0	0.01852	0	0	0.02273	0	0.11111	0	0.02083
	**192**	0	0	0	0	0	0	0	0	0	0	0	0	0	0	0	0	0	0	0	0
	**193**	0	0	0	0	0	0	0	0	0	0	0	0	0	0	0	0	0	0	0	0
	**194**	0	0	0	0	0	0	0	0	0	0	0	0	0	0.04167	0	0	0	0	0	0
	**195**	0	0	0	0	0	0.05556	0	0	0	0	0	0	0	0	0	0	0	0	0	0
	**197**	0	0	0	0	0	0	0	0	0	0	0	0	0	0	0	0	0	0	0	0
	**198**	0	0.25	0	0	0	0	0	0	0	0	0.08333	0	0	0	0	0	0	0	0	0
	**199**	0	0	0	0	0	0	0	0	0	0	0	0	0	0	0	0	0	0	0	0.04167
	**201**	0	0	0	0	0	0	0	0	0	0	0	0	0	0	0	0.02273	0	0	0	0.02083
	**202**	0.4	0	0	0.03125	0	0	0	0	0.04762	0	0	0	0.03704	0	0	0	0	0	0	0
	**203**	0	0	0	0	0.5	0.11111	0	0	0.04762	0	0.25	0.09091	0.01852	0.04167	0.4	0.11364	0	0.05556	0.09091	0.125
	**204**	0	0	0	0	0	0	0	0	0	0	0	0	0	0	0	0	0	0	0	0
	**205**	0	0	0	0	0	0	0	0	0	0	0	0.04545	0	0	0	0	0	0	0	0
	**206**	0	0	0.025	0	0	0	0	0	0	0	0	0	0	0	0	0	0	0	0	0
	**207**	0	0	0	0	0	0	0	0	0	0	0	0	0	0	0	0	0	0	0	0
	**208**	0	0.25	0	0	0	0.05556	0	0	0	0	0.08333	0.09091	0.01852	0.04167	0	0	0	0	0	0
	**209**	0	0	0	0	0	0	0	0	0	0	0	0	0	0	0	0	0	0	0	0.04167
	**210**	0	0	0.125	0	0	0.16667	0	0	0.19048	0	0	0	0.03704	0.08333	0	0.09091	0.125	0.11111	0.06061	0
	**212**	0	0	0.275	0.3125	0	0.16667	0	0.25	0.2619	0.28571	0.16667	0.18182	0.37037	0.20833	0.2	0.36364	0.4375	0.16667	0.43939	0.25
	**214**	0	0	0.275	0	0.5	0.11111	0	0	0.02381	0.07143	0	0.04545	0.07407	0	0	0.13636	0.0625	0.11111	0.06061	0.0625
	**215**	0	0	0	0	0	0	0	0	0	0	0	0	0	0	0	0	0	0	0	0
	**216**	0.3	0	0.1	0.15625	0	0	1	0.5	0.02381	0.21429	0.41667	0.22727	0.22222	0.29167	0.2	0.11364	0.3125	0.33333	0.24242	0.20833
	**217**	0	0	0	0	0	0	0	0	0	0	0	0	0	0	0	0	0	0	0	0.02083
	**218**	0	0	0.075	0	0	0	0	0	0	0	0	0	0	0	0	0	0	0	0	0
	**219**	0	0	0	0	0	0.11111	0	0	0.02381	0.28571	0	0	0	0.08333	0	0	0	0	0	0.02083
	**220**	0	0	0	0	0	0	0	0	0.07143	0	0	0	0	0	0	0	0.0625	0	0	0.02083
	**221**	0	0	0	0	0	0	0	0	0	0	0	0	0	0	0	0	0	0	0	0.02083
	**222**	0	0	0	0	0	0.05556	0	0	0	0	0	0	0	0	0	0	0	0	0	0
	**224**	0	0	0	0	0	0	0	0	0	0	0	0	0	0	0	0	0	0	0	0
	**225**	0	0	0	0	0	0	0	0	0	0	0	0	0	0	0	0	0	0	0	0

Table S7. Continued.

	Study sites																				
**Locus**	**Allele/n**	**TH BAN**	**TH CHA**	**TH DA1**	**TH DA2**	**KH PNP**	**IN DEL**	**NP POK**	**PH BUS**	**PH GUG**	**PH GUZ**	**PH LU1**	**PH LU2**	**PH LU3**	**PH MIN**	**PH PA1**	**PH PA2**	**PH PA3**	**PH PA4**	**PH PA5**	**MY PEN**
**Bi7**	**N**	5	4	19	13	1	7	1	4	21	8	6	9	28	13	5	21	8	9	33	24
	**101**	0	0	0	0	0	0	0	0	0	0	0	0	0	0	0	0	0	0	0	0
	**103**	0	0	0	0	0	0	0	0	0	0	0	0	0	0	0	0	0	0	0	0
	**107**	0	0	0	0	0	0	0	0	0	0	0	0	0	0	0	0	0	0	0	0
	**108**	0	0	0	0	0	0	0	0	0	0	0	0	0	0	0	0	0	0	0	0
	**109**	0	0	0	0	0	0	0	0.25	0	0.0625	0	0.05556	0	0.19231	0	0.02381	0.0625	0.11111	0.06061	0.02083
	**115**	0	0	0	0	0	0	0	0	0	0	0	0	0.05357	0	0	0	0.0625	0	0	0
	**116**	0	0	0	0	0	0	0	0	0	0	0	0	0	0	0	0	0	0	0	0
	**117**	0	0	0	0	0	0	0	0	0	0	0	0.05556	0	0.03846	0	0	0	0.05556	0.01515	0
	**118**	0	0	0	0	0	0	0	0	0	0	0	0	0	0	0	0	0	0	0.13636	0
	**119**	0	0	0.10526	0.03846	0	0.07143	0	0.25	0	0.0625	0.25	0.22222	0.26786	0.07692	0.1	0.04762	0.0625	0	0.10606	0.04167
	**120**	0	0	0	0	0	0	0	0	0	0	0	0	0.05357	0	0	0	0	0	0.01515	0.02083
	**121**	0.3	0	0	0	0	0.14286	0	0	0	0	0	0	0	0.03846	0	0	0	0	0.01515	0
	**122**	0	0	0	0	0	0	0	0	0	0	0	0	0.01786	0	0	0	0	0	0.01515	0.04167
	**123**	0	0	0.02632	0	0	0	0	0	0	0	0	0	0	0	0	0	0	0	0	0
	**125**	0	0	0.10526	0.07692	0	0	0	0	0.04762	0	0	0.05556	0	0	0	0.02381	0	0	0	0
	**126**	0	0	0	0	0	0	0	0	0	0	0	0	0	0	0	0	0	0	0	0
	**127**	0	0	0.05263	0	0	0	0	0	0	0	0	0	0.01786	0	0	0.02381	0	0	0	0.125
	**128**	0	0	0	0	0	0	0	0	0	0	0	0	0	0	0	0	0	0	0	0
	**129**	0	0	0.10526	0.19231	0	0.14286	0	0	0	0.0625	0	0.05556	0.05357	0.07692	0.1	0.11905	0.125	0.11111	0.12121	0.14583
	**130**	0	0	0	0	0	0	0	0	0	0	0	0	0	0	0	0	0	0	0	0
	**131**	0.1	0.375	0.28947	0.19231	0	0.14286	0.5	0.25	0.16667	0.5	0	0.16667	0.14286	0.30769	0	0.04762	0.0625	0.27778	0.13636	0.25
	**133**	0.5	0.625	0.18421	0.46154	1	0.35714	0.5	0.25	0.42857	0.25	0.66667	0.16667	0.33929	0.26923	0.2	0.2619	0.25	0.27778	0.24242	0.33333
	**134**	0	0	0	0	0	0	0	0	0	0	0	0	0	0	0.1	0	0	0	0	0
	**135**	0	0	0	0	0	0	0	0	0	0	0	0	0	0	0	0	0	0	0	0.02083
	**136**	0.1	0	0.05263	0.03846	0	0	0	0	0	0.0625	0	0	0.03571	0	0.3	0	0.0625	0.05556	0.01515	0
	**138**	0	0	0.07895	0	0	0	0	0	0.02381	0	0	0	0	0	0	0	0	0	0	0
	**140**	0	0	0	0	0	0	0	0	0.02381	0	0	0	0	0	0	0.02381	0	0	0	0
	**141**	0	0	0	0	0	0	0	0	0	0	0	0	0	0	0	0	0	0	0	0
	**142**	0	0	0	0	0	0	0	0	0	0	0	0.05556	0	0	0	0	0.125	0	0	0
	**143**	0	0	0	0	0	0	0	0	0	0	0	0	0.01786	0	0	0	0.0625	0	0.0303	0
	**144**	0	0	0	0	0	0.14286	0	0	0.30952	0	0.08333	0.16667	0	0	0.2	0.42857	0.125	0.11111	0.09091	0
**Locus**	**Allele/n**	**TH BAN**	**TH CHA**	**TH DA1**	**TH DA2**	**KH PNP**	**IN DEL**	**NP POK**	**PH BUS**	**PH GUG**	**PH GUZ**	**PH LU1**	**PH LU2**	**PH LU3**	**PH MIN**	**PH PA1**	**PH PA2**	**PH PA3**	**PH PA4**	**PH PA5**	**MY PEN**
**Bt9.1a**	**N**	5	4	20	16	1	9	1	4	24	8	6	12	28	13	6	22	8	9	33	24
	**108**	0	0	0	0	0	0	0	0	0	0	0	0	0	0	0	0	0	0	0	0
	**115**	0	0	0	0	0	0	0	0	0	0	0.08333	0.04167	0.125	0	0	0	0	0	0	0
	**117**	0	0	0	0	0	0	0	0	0	0	0	0	0	0	0	0	0	0	0	0
	**118**	0	0	0	0	0	0	0	0	0	0	0	0	0	0	0	0	0	0	0	0
	**119**	0.2	0	0.025	0	0	0	0	0	0	0	0	0	0	0	0	0	0	0	0	0
	**120**	0	0	0	0	0	0.11111	0	0	0	0	0	0	0	0	0	0	0	0	0	0.02083
	**121**	0.1	0	0.025	0	0	0	0	0	0	0	0	0	0	0	0	0	0	0	0	0
	**122**	0	0	0	0	0	0	0	0	0	0	0	0	0	0	0	0	0	0	0	0.02083
	**123**	0	0	0	0	0	0	0	0	0	0	0	0	0	0	0	0	0	0	0	0
	**125**	0	0	0.05	0.03125	0	0	0	0	0.04167	0	0	0	0	0	0	0	0	0	0	0
	**126**	0	0	0	0	0	0	0	0	0	0	0	0	0	0	0	0.02273	0	0	0	0
	**127**	0	0	0	0.03125	0	0	0	0	0	0.0625	0	0	0.03571	0	0	0	0	0	0	0
	**128**	0	0	0	0	0	0	0	0	0	0	0	0	0	0	0	0	0	0	0	0.14583
	**129**	0.1	0.125	0.1	0.1875	0	0.22222	0	0	0.08333	0.0625	0	0.04167	0.08929	0.19231	0.41667	0.18182	0.25	0.11111	0.21212	0.14583
	**130**	0	0	0	0	0	0	0	0	0	0	0	0	0	0	0	0	0	0	0	0
	**131**	0.2	0.375	0.45	0.40625	0.5	0.22222	0	0.375	0.16667	0.5625	0	0.33333	0.21429	0.38462	0.08333	0.04545	0.25	0.33333	0.21212	0.25
	**133**	0.4	0.5	0.25	0.3125	0.5	0.44444	1	0.625	0.64583	0.3125	0.91667	0.54167	0.44643	0.42308	0.33333	0.68182	0.5	0.55556	0.56061	0.375
	**134**	0	0	0	0	0	0	0	0	0	0	0	0	0	0	0	0	0	0	0	0
	**135**	0	0	0.05	0	0	0	0	0	0.04167	0	0	0	0	0	0.08333	0	0	0	0	0.04167
	**136**	0	0	0	0	0	0	0	0	0	0	0	0	0	0	0	0	0	0	0	0
	**137**	0	0	0.025	0.03125	0	0	0	0	0	0	0	0.04167	0	0	0.08333	0.02273	0	0	0	0

	138	0	0	0	0	0	0	0	0	0	0	0	0	0	0	0	0	0	0	0	0
	139	0	0	0	0	0	0	0	0	0	0	0	0	0	0	0	0	0	0	0	0
	140	0	0	0	0	0	0	0	0	0	0	0	0	0	0	0	0.02273	0	0	0	0
	142	0	0	0	0	0	0	0	0	0	0	0	0	0	0	0	0.02273	0	0	0	0
	144	0	0	0.025	0	0	0	0	0	0.02083	0	0	0	0.03571	0	0	0	0	0	0.01515	0
	146	0	0	0	0	0	0	0	0	0	0	0	0	0.01786	0	0	0	0	0	0	0
	148	0	0	0	0	0	0	0	0	0	0	0	0	0.01786	0	0	0	0	0	0	0
	151	0	0	0	0	0	0	0	0	0	0	0	0	0.01786	0	0	0	0	0	0	0

Table S7. Continued.

	**Study sites**																				
**Locus**	**Allele/n**	**TH BAN**	**TH CHA**	**TH DA1**	**TH DA2**	**KH PNP**	**IN DEL**	**NP POK**	**PH BUS**	**PH GUG**	**PH GUZ**	**PH LU1**	**PH LU2**	**PH LU3**	**PH MIN**	**PH PA1**	**PH PA2**	**PH PA3**	**PH PA4**	**PH PA5**	**MY PEN**
**Bt7.2b**	**N**	5	4	20	16	1	9	1	4	24	8	6	13	28	13	6	22	8	9	33	24
	**102**	0	0	0	0	0	0	0	0	0	0	0	0	0	0	0	0	0	0	0	0
	**105**	0	0	0	0	0	0	0	0	0	0	0	0	0	0	0	0	0	0	0	0
	**109**	0	0	0	0	0	0	0	0	0	0	0	0	0	0	0	0	0	0	0	0
	**123**	0	0	0	0	0	0	0	0	0	0	0	0	0	0	0	0	0	0	0	0
	**131**	0	0	0	0	0	0	0	0	0	0	0	0	0	0	0	0	0	0	0	0
	**133**	0	0	0	0	0	0	0	0	0	0	0	0	0	0.03846	0	0	0	0	0	0
	**135**	0	0	0	0.0625	0	0	0	0	0	0	0	0	0	0	0	0	0	0	0	0.02083
	**138**	0	0	0	0	0	0	0	0	0	0	0	0	0	0	0	0	0	0	0	0
	**139**	0	0	0	0	0	0	0	0	0	0	0	0	0	0	0	0	0	0	0	0
	**140**	0	0	0	0	0	0	0	0	0	0	0	0	0.07143	0.07692	0	0	0	0	0	0
	**141**	0.4	0.5	0.65	0.53125	0	0	0	0	0.04167	0	0.16667	0	0.07143	0	0	0	0	0	0	0.25
	**142**	0	0	0	0	0	0.38889	0	0.375	0.29167	0.0625	0.25	0.34615	0.08929	0.07692	0	0.09091	0.1875	0.16667	0.09091	0
	**143**	0.4	0.5	0.325	0.375	0	0.16667	0	0	0	0.25	0.08333	0.03846	0.28571	0.34615	0.16667	0.09091	0.125	0.05556	0.25758	0.58333
	**144**	0	0	0	0	1	0.44444	1	0.375	0.60417	0.4375	0.5	0.5	0.33929	0.38462	0.75	0.81818	0.625	0.61111	0.59091	0.10417
	**145**	0	0	0	0	0	0	0	0	0	0	0	0	0	0	0	0	0	0	0	0.02083
	**146**	0	0	0	0	0	0	0	0	0.02083	0	0	0	0	0	0	0	0	0	0	0
	**147**	0	0	0	0.03125	0	0	0	0	0	0	0	0	0	0	0	0	0	0	0	0
	**148**	0	0	0	0	0	0	0	0	0	0.0625	0	0.07692	0.01786	0.03846	0	0	0	0.05556	0	0
	**149**	0	0	0	0	0	0	0	0	0	0	0	0	0	0	0	0	0	0	0	0
	**150**	0	0	0	0	0	0	0	0	0	0	0	0	0.01786	0	0	0	0	0	0	0
	**151**	0.2	0	0.025	0	0	0	0	0.25	0.04167	0.1875	0	0.03846	0.03571	0.03846	0.08333	0	0.0625	0.11111	0.06061	0
	**153**	0	0	0	0	0	0	0	0	0	0	0	0	0	0	0	0	0	0	0	0.02083
	**163**	0	0	0	0	0	0	0	0	0	0	0	0	0	0	0	0	0	0	0	0
	**167**	0	0	0	0	0	0	0	0	0	0	0	0	0	0	0	0	0	0	0	0
	**171**	0	0	0	0	0	0	0	0	0	0	0	0	0	0	0	0	0	0	0	0
	**182**	0	0	0	0	0	0	0	0	0	0	0	0	0.03571	0	0	0	0	0	0	0
	**183**	0	0	0	0	0	0	0	0	0	0	0	0	0	0	0	0	0	0	0	0
	**187**	0	0	0	0	0	0	0	0	0	0	0	0	0.03571	0	0	0	0	0	0	0
	**189**	0	0	0	0	0	0	0	0	0	0	0	0	0	0	0	0	0	0	0	0
**Locus**	**Allele/n**	**TH BAN**	**TH CHA**	**TH DA1**	**TH DA2**	**KH PNP**	**IN DEL**	**NP POK**	**PH BUS**	**PH GUG**	**PH GUZ**	**PH LU1**	**PH LU2**	**PH LU3**	**PH MIN**	**PH PA1**	**PH PA2**	**PH PA3**	**PH PA4**	**PH PA5**	**MY PEN**
**Bi11**	**N**	5	4	20	16	1	9	1	4	24	8	6	13	28	13	6	22	8	9	33	24
	**108**	0	0	0	0	0	0.11111	0	0	0.0625	0.0625	0.16667	0.07692	0.03571	0.03846	0.25	0.18182	0.1875	0.11111	0.06061	0
	**110**	0	0	0	0	0	0	0	0	0	0	0	0	0	0	0	0	0	0	0	0
	**120**	0	0	0	0	0	0.05556	0	0	0	0	0	0	0	0	0	0	0	0	0	0
	**123**	0	0	0	0	0	0	0	0	0	0	0	0	0	0	0	0	0	0	0	0
	**126**	0	0	0	0	0	0	0	0	0	0	0	0	0	0	0	0.02273	0	0	0	0
	**127**	0	0	0	0	0	0	0	0	0	0	0	0.07692	0	0	0	0	0	0	0	0
	**129**	0	0	0	0	0	0.05556	0	0	0.02083	0	0	0	0	0.03846	0	0.04545	0.0625	0.05556	0.10606	0
	**131**	0	0	0	0	0.5	0	0	0	0	0.125	0	0.03846	0.03571	0.07692	0	0	0.0625	0.05556	0.10606	0
	**133**	0	0	0	0	0.5	0.11111	0.5	0.25	0.04167	0.25	0.33333	0.07692	0.16071	0.26923	0	0.09091	0	0.05556	0.18182	0
	**134**	0	0	0	0	0	0	0	0	0	0	0	0	0	0	0	0	0	0	0	0.02083
	**136**	0	0	0	0	0	0	0	0	0	0	0	0	0.01786	0	0	0	0	0	0	0
	**138**	0	0	0	0	0	0	0	0	0	0	0	0	0	0	0	0	0	0	0	0
	**140**	0	0	0.075	0	0	0	0	0	0	0	0	0	0	0	0	0.02273	0	0	0	0
	**142**	0.3	0.25	0	0.125	0	0	0	0	0	0	0	0	0	0	0	0	0	0	0	0
	**143**	0	0	0	0	0	0.44444	0.5	0.25	0.08333	0.0625	0.16667	0.34615	0.125	0.03846	0.25	0.22727	0.3125	0.16667	0.18182	0

**144**	0	0	0	0.0625	0	0	0	0	0	0	0.08333	0	0.01786	0	0	0	0	0	0	0.02083
**145**	0	0	0	0	0	0.16667	0	0.25	0.0625	0	0.16667	0.15385	0.01786	0.07692	0.16667	0.15909	0.125	0.11111	0.12121	0
**146**	0	0.125	0	0.09375	0	0	0	0	0.02083	0	0	0	0	0	0	0	0	0	0	0
**148**	0.1	0	0	0	0	0	0	0	0	0	0	0	0.01786	0	0	0	0	0.05556	0	0
**149**	0	0	0	0	0	0	0	0	0	0	0	0	0	0	0	0	0	0	0	0
**150**	0	0	0	0	0	0.05556	0	0	0.08333	0.125	0	0	0.01786	0	0	0.04545	0	0	0	0
**152**	0.6	0.625	0.925	0.6875	0	0	0	0.25	0.5625	0.375	0.08333	0.23077	0.51786	0.46154	0.33333	0.20455	0.25	0.38889	0.24242	0.95833
**153**	0	0	0	0	0	0	0	0	0.0625	0	0	0	0	0	0	0	0	0	0	0
**154**	0	0	0	0.03125	0	0	0	0	0	0	0	0	0	0	0	0	0	0	0	0
**156**	0	0	0	0	0	0	0	0	0	0	0	0	0	0	0	0	0	0	0	0
**159**	0	0	0	0	0	0	0	0	0	0	0	0	0	0	0	0	0	0	0	0
**161**	0	0	0	0	0	0	0	0	0	0	0	0	0	0	0	0	0	0	0	0
**166**	0	0	0	0	0	0	0	0	0	0	0	0	0	0	0	0	0	0	0	0
**183**	0	0	0	0	0	0	0	0	0	0	0	0	0.03571	0	0	0	0	0	0	0

Table S7. Continued.

	Study sites																				
**Locus**	**Allele/n**	**TH BAN**	**TH CHA**	**TH DA1**	**TH DA2**	**KH PNP**	**IN DEL**	**NP POK**	**PH BUS**	**PH GUG**	**PH GUZ**	**PH LU1**	**PH LU2**	**PH LU3**	**PH MIN**	**PH PA1**	**PH PA2**	**PH PA3**	**PH PA4**	**PH PA5**	**MY PEN**
**Bp181**	**N**	5	4	18	15	1	9	1	3	23	8	6	13	28	13	6	22	8	9	33	24
	**108**	0	0	0	0	0	0	0	0	0	0	0	0	0	0	0	0	0	0	0	0
	**110**	0	0	0	0	0	0.05556	0	0	0	0	0	0	0	0	0	0	0	0	0	0
	**114**	0	0	0	0.06667	0	0	0	0	0.02174	0	0	0	0	0	0	0	0	0	0	0
	**116**	0	0	0	0	0	0	0	0	0	0	0	0	0	0	0	0	0	0	0	0
	**117**	0	0	0	0	0	0	0	0	0.02174	0	0	0	0	0	0	0	0	0	0.01515	0
	**118**	0	0.125	0.22222	0.1	0	0.22222	0	0	0	0	0	0	0.05357	0	0	0	0	0	0	0
	**119**	0	0	0	0	0	0	0	0	0	0	0	0	0	0	0	0	0	0	0	0
	**120**	0.8	0.375	0.52778	0.4	0	0.16667	0.5	0.16667	0.3913	0.375	0.5	0.57692	0.58929	0.61538	0.5	0.5	0.5	0.38889	0.39394	0.39583
	**121**	0	0	0	0	0	0.33333	0	0.33333	0.08696	0.25	0.08333	0	0.01786	0	0	0	0	0.22222	0.06061	0.29167
	**122**	0.2	0.25	0.22222	0.26667	1	0.22222	0.5	0.5	0.13043	0.375	0.33333	0.38462	0.26786	0.38462	0.5	0.40909	0.5	0.33333	0.5	0.3125
	**123**	0	0	0	0	0	0	0	0	0.02174	0	0.08333	0.03846	0.03571	0	0	0	0	0	0	0
	**124**	0	0	0.02778	0	0	0	0	0	0.02174	0	0	0	0	0	0	0	0	0	0	0
	**126**	0	0	0	0	0	0	0	0	0.02174	0	0	0	0	0	0	0	0	0	0	0
	**127**	0	0	0	0	0	0	0	0	0	0	0	0	0	0	0	0	0	0.05556	0	0
	**130**	0	0.125	0	0.1	0	0	0	0	0	0	0	0	0	0	0	0	0	0	0	0
	**132**	0	0.125	0	0.03333	0	0	0	0	0	0	0	0	0	0	0	0	0	0	0	0
	**136**	0	0	0	0	0	0	0	0	0	0	0	0	0	0	0	0	0	0	0	0
	**138**	0	0	0	0	0	0	0	0	0	0	0	0	0	0	0	0	0	0	0	0
	**141**	0	0	0	0	0	0	0	0	0	0	0	0	0	0	0	0	0	0	0	0
	**146**	0	0	0	0.03333	0	0	0	0	0	0	0	0	0	0	0	0	0	0	0	0
	**148**	0	0	0	0	0	0	0	0	0.28261	0	0	0	0.03571	0	0	0.09091	0	0	0.0303	0
	**191**	0	0	0	0	0	0	0	0	0	0	0	0	0	0	0	0	0	0	0	0
**Locus**	**Allele/n**	**TH BAN**	**TH CHA**	**TH DA1**	**TH DA2**	**KH PNP**	**IN DEL**	**NP POK**	**PH BUS**	**PH GUG**	**PH GUZ**	**PH LU1**	**PH LU2**	**PH LU3**	**PH MIN**	**PH PA1**	**PH PA2**	**PH PA3**	**PH PA4**	**PH PA5**	**MY PEN**
**Bt4.6a**	**N**	5	4	18	16	1	9	1	4	23	8	6	13	28	13	6	22	8	9	33	24
	**114**	0	0	0	0	0	0	0	0	0	0.0625	0	0	0	0	0	0	0	0	0	0
	**116**	0	0	0	0	0	0	0	0	0	0	0	0	0	0	0	0	0	0	0	0
	**118**	0	0	0	0	0	0	0	0	0	0	0	0	0	0	0	0	0	0	0	0
	**119**	0	0	0	0	0	0	0	0	0	0	0	0	0	0	0	0	0	0	0	0
	**120**	0	0	0	0	0	0	0	0	0	0	0	0	0	0	0	0	0	0	0	0
	**121**	0	0	0	0	0	0	0	0	0	0	0	0	0	0	0	0	0	0	0	0
	**122**	0	0	0	0	0	0	0	0	0	0	0	0	0	0	0	0	0	0	0	0
	**125**	0	0	0	0.0625	0	0	0	0.25	0	0	0	0	0	0	0	0	0	0	0	0
	**127**	0	0	0	0	0	0	0	0	0	0	0	0	0	0	0	0	0	0	0	0
	**129**	0	0	0	0	0	0.05556	0	0	0	0	0	0	0	0	0	0	0	0	0	0
	**131**	0	0	0	0	0	0	0	0	0	0	0	0	0	0	0	0	0	0	0	0
	**132**	0	0	0	0	0	0	0	0	0	0	0	0	0	0	0	0	0	0	0	0
	**133**	0	0	0	0	0	0	0	0	0	0	0	0	0.01786	0	0	0	0	0	0	0
	**135**	0	0	0	0	0	0	0	0	0	0	0	0	0	0	0	0	0	0	0.01515	0
	**136**	0	0	0	0	0	0	0	0	0	0	0	0	0	0	0	0	0	0	0	0
	**137**	0	0.125	0.02778	0	0	0	0	0	0.02174	0	0	0.03846	0.03571	0	0	0.02273	0	0	0.01515	0.02083
	**139**	0.1	0.125	0.11111	0.0625	0.5	0.11111	0	0	0.02174	0	0	0	0	0	0	0.02273	0	0	0	0.0625
	**141**	0.2	0	0.05556	0.34375	0	0.11111	0	0	0.02174	0	0.33333	0.07692	0.03571	0.19231	0.08333	0	0.125	0	0.0303	0.08333
	**143**	0.1	0	0.19444	0.09375	0	0	0	0	0	0	0	0	0.01786	0	0	0	0.0625	0	0	0
	**144**	0	0	0	0	0	0	0	0.125	0.02174	0.125	0.08333	0.19231	0.14286	0.11538	0.08333	0.13636	0.1875	0.33333	0.04545	0.04167
	**145**	0.2	0.375	0.11111	0.3125	0	0	0	0	0.02174	0	0.08333	0.03846	0.05357	0.03846	0	0	0.0625	0	0	0
	**146**	0	0	0	0	0	0.05556	0.5	0.5	0.02174	0.375	0.5	0.30769	0.25	0.23077	0.5	0.29545	0.25	0.22222	0.5	0.08333
	**147**	0.1	0	0.05556	0.03125	0	0	0	0	0	0	0	0	0	0	0	0	0	0	0	0
	**148**	0	0.125	0.02778	0	0	0.16667	0	0	0.73913	0.0625	0	0.23077	0.35714	0.07692	0.33333	0.38636	0.125	0.27778	0.22727	0.04167
	**149**	0	0	0	0.03125	0	0	0	0	0	0	0	0	0	0	0	0	0	0	0	0
	**150**	0.1	0	0.05556	0	0	0	0	0	0.04348	0.0625	0	0.03846	0	0.03846	0	0	0	0	0	0
	**151**	0	0	0	0	0.5	0.33333	0	0	0	0.1875	0	0.03846	0	0.07692	0	0.09091	0.1875	0.05556	0.07576	0.02083
	**152**	0	0.125	0	0	0	0	0	0	0	0.0625	0	0	0.03571	0	0	0	0	0	0	0
	**153**	0	0	0.08333	0	0	0	0	0	0.08696	0	0	0.03846	0	0.03846	0	0	0	0.05556	0.0303	0
	**154**	0.1	0.125	0.08333	0	0	0	0	0.125	0	0.0625	0	0	0.01786	0.15385	0	0.04545	0	0.05556	0.01515	0.60417
	**155**	0	0	0.13889	0.03125	0	0	0	0	0	0	0	0	0	0	0	0	0	0	0	0.02083
	**156**	0.1	0	0.02778	0	0	0.05556	0	0	0	0	0	0	0.01786	0.03846	0	0	0	0	0.01515	0
	**157**	0	0	0.02778	0	0	0	0	0	0	0	0	0	0	0	0	0	0	0	0	0
	**158**	0	0	0	0	0	0.05556	0.5	0	0	0	0	0	0	0	0	0	0	0	0.01515	0
	**159**	0	0	0	0	0	0	0	0	0	0	0	0	0	0	0	0	0	0	0	0
	**160**	0	0	0	0	0	0	0	0	0	0	0	0	0	0	0	0	0	0	0	0
	**161**	0	0	0	0	0	0.05556	0	0	0	0	0	0	0	0	0	0	0	0	0.01515	0
	**162**	0	0	0	0	0	0	0	0	0	0	0	0	0	0	0	0	0	0	0	0
	**163**	0	0	0	0	0	0	0	0	0	0	0	0	0	0	0	0	0	0	0	0.02083

	**164**	0	0	0	0	0	0	0	0	0	0	0	0	0.01786	0	0	0	0	0	0	0
	**165**	0	0	0	0.03125	0	0	0	0	0	0	0	0	0	0	0	0	0	0	0	0

Table S7. Continued.

	**Study sites**																				
**Locus**	**Allele/n**	**TH BAN**	**TH CHA**	**TH DA1**	**TH DA2**	**KH PNP**	**IN DEL**	**NP POK**	**PH BUS**	**PH GUG**	**PH GUZ**	**PH LU1**	**PH LU2**	**PH LU3**	**PH MIN**	**PH PA1**	**PH PA2**	**PH PA3**	**PH PA4**	**PH PA5**	**MY PEN**
**Boms60**	**N**	5	4	18	16	1	9	1	3	24	8	6	13	28	13	6	22	8	9	33	24
	**128**	0	0	0	0	0	0	0	0	0	0	0	0	0	0	0	0	0	0	0	0
	**138**	0	0	0	0	0	0	0	0	0	0	0	0	0	0	0	0	0	0	0	0
	**153**	0	0	0	0	0	0	0	0	0	0	0	0	0.01786	0	0	0	0	0	0	0
	**155**	0	0	0	0	0	0	0	0	0	0	0	0	0	0	0	0	0	0	0	0
	**156**	0	0	0	0	0	0	0	0	0.02083	0	0	0	0.01786	0	0	0	0	0	0	0
	**161**	0	0	0	0.0625	0	0	0	0	0	0	0	0	0	0	0	0	0	0	0	0
	**163**	0	0	0	0	0	0	0	0	0.04167	0	0	0	0.01786	0	0.08333	0	0	0	0	0
	**164**	0	0	0	0	0	0	0	0	0.04167	0	0	0	0	0	0.08333	0.02273	0	0	0	0
	**169**	0	0	0	0.09375	0	0	0	0	0	0	0	0	0	0	0	0.02273	0	0	0	0
	**170**	0	0	0	0	0	0	0	0	0	0	0	0	0	0	0	0	0	0	0	0.02083
	**171**	0	0	0	0	0	0	0	0	0	0	0	0	0	0	0	0	0	0	0	0.02083
	**173**	0	0	0	0	0	0	0	0	0	0	0	0	0	0	0	0	0	0	0	0
	**174**	0	0	0	0.0625	0	0	0	0	0	0	0	0	0	0	0	0	0	0	0	0
	**176**	0	0	0	0	0	0	0	0	0	0	0	0.03846	0	0	0	0	0	0	0	0
	**177**	0	0.125	0	0	0	0	0	0	0	0	0	0	0	0	0	0	0.1875	0	0	0
	**178**	0	0	0	0.03125	0	0	0	0	0.02083	0.0625	0	0	0	0	0	0	0	0	0	0
	**179**	0	0	0	0	0	0	0	0	0.02083	0	0	0	0	0	0	0	0	0	0	0.02083
	**180**	0.1	0.25	0.11111	0.03125	0	0.05556	0	0	0	0	0	0	0.07143	0	0	0	0.125	0	0	0.08333
	**181**	0	0	0.02778	0.09375	0.5	0.27778	1	0	0.14583	0.0625	0.41667	0.15385	0.14286	0.26923	0.08333	0.06818	0	0.11111	0.12121	0.29167
	**182**	0.5	0.25	0.19444	0.40625	0	0.33333	0	0	0.20833	0.125	0	0.19231	0.08929	0.19231	0.08333	0.15909	0.1875	0.33333	0.15152	0.14583
	**183**	0.2	0.25	0.27778	0	0	0.11111	0	0	0.29167	0.1875	0	0.07692	0.03571	0.07692	0.08333	0.15909	0.0625	0	0.13636	0.14583
	**184**	0	0	0.16667	0	0.5	0.16667	0	0.16667	0.125	0.25	0.08333	0.07692	0.17857	0.15385	0.08333	0.18182	0.1875	0	0.10606	0.08333
	**185**	0	0.125	0.05556	0.03125	0	0	0	0	0	0.0625	0	0.19231	0.07143	0.15385	0.16667	0.04545	0.125	0.05556	0.01515	0.10417
	**186**	0.1	0	0.05556	0	0	0	0	0.16667	0.02083	0.1875	0.25	0.07692	0.08929	0.07692	0.08333	0.04545	0	0.22222	0.06061	0.02083
	**187**	0	0	0.02778	0	0	0.05556	0	0.5	0.02083	0	0.08333	0.07692	0.16071	0	0	0.04545	0.125	0	0.09091	0.02083
	**188**	0	0	0.02778	0.125	0	0	0	0.16667	0.04167	0.0625	0.16667	0.11538	0.08929	0.07692	0.08333	0.20455	0	0.16667	0.27273	0.02083
	**189**	0.1	0	0.02778	0.03125	0	0	0	0	0	0	0	0	0	0	0	0.02273	0	0.05556	0.0303	0
	**190**	0	0	0.02778	0.03125	0	0	0	0	0	0	0	0	0.01786	0	0.16667	0.02273	0	0.05556	0.01515	0
	**191**	0	0	0	0	0	0	0	0	0	0	0	0	0	0	0	0	0	0	0	0
	**192**	0	0	0	0	0	0	0	0	0	0	0	0	0	0	0	0	0	0	0	0
	**195**	0	0	0	0	0	0	0	0	0	0	0	0	0	0	0	0	0	0	0	0.02083

Table S7. Continued.

	Study sites																				
**Locus**	**Allele/n**	**TH BAN**	**TH CHA**	**TH DA1**	**TH DA2**	**KH PNP**	**IN DEL**	**NP POK**	**PH BUS**	**PH GUG**	**PH GUZ**	**PH LU1**	**PH LU2**	**PH LU3**	**PH MIN**	**PH PA1**	**PH PA2**	**PH PA3**	**PH PA4**	**PH PA5**	**MY PEN**
**Bt5**	**N**	5	4	19	15	1	9	1	4	24	8	6	13	28	13	6	22	8	9	33	24
	**139**	0	0	0	0	0	0	0	0	0	0	0	0	0	0	0	0	0	0	0	0
	**146**	0	0	0	0.03333	0	0	0	0	0	0	0	0	0	0	0	0	0	0	0	0
	**148**	0	0	0	0	0	0	0	0	0.20833	0	0	0	0.03571	0	0	0.06818	0	0	0	0
	**150**	0	0	0	0	0	0	0	0	0	0	0	0	0	0	0	0	0	0	0	0
	**151**	0.1	0	0.07895	0.03333	0	0	0	0	0	0	0	0	0.07143	0	0	0	0	0	0	0
	**152**	0	0	0	0	0	0	0	0	0	0	0	0.07692	0	0.03846	0	0.04545	0.0625	0	0.07576	0
	**153**	0	0	0	0	0	0	0	0	0.0625	0	0	0	0	0.07692	0	0	0	0	0.01515	0
	**154**	0	0	0.02632	0	0	0	0	0	0	0	0	0	0	0	0	0	0	0	0	0
	**155**	0	0	0.13158	0	0	0	0	0.25	0	0	0	0	0	0.07692	0	0.04545	0	0	0	0
	**157**	0	0	0	0	0	0	0	0	0	0	0	0	0	0	0	0	0	0	0.0303	0
	**159**	0	0	0	0	0	0	0	0	0	0	0	0.03846	0	0	0	0	0.0625	0.16667	0	0
	**160**	0	0	0	0	0	0	0	0	0	0	0	0	0	0	0	0	0	0	0	0
	**163**	0	0	0	0	0	0	0	0	0.04167	0	0	0	0.01786	0	0.16667	0	0	0	0	0
	**166**	0	0	0	0	0	0	0	0	0	0	0	0	0	0	0	0	0	0	0	0
	**168**	0	0	0	0	0	0	0	0	0	0	0	0	0	0	0	0	0	0	0	0
	**169**	0	0	0	0	0	0	0	0	0	0	0	0	0	0	0	0	0	0	0	0
	**170**	0	0	0	0	0	0	0	0	0.02083	0.125	0.08333	0.07692	0.03571	0	0	0.13636	0.0625	0	0.01515	0.02083
	**171**	0	0	0	0.06667	0	0	0	0	0	0	0	0	0	0	0.08333	0.04545	0	0	0	0
	**172**	0	0	0	0	1	0.22222	0	0	0.33333	0.25	0	0	0.125	0.19231	0.33333	0.34091	0.125	0.16667	0.30303	0.5625
	**173**	0	0	0	0	0	0	0	0	0.08333	0	0.16667	0.07692	0.05357	0.03846	0	0.06818	0.1875	0.05556	0.04545	0.02083
	**175**	0	0	0	0.1	0	0	0	0	0	0	0.16667	0	0	0	0	0	0	0	0	0
	**176**	0	0	0	0	0	0	0	0	0	0	0	0	0	0	0	0	0	0	0	0
	**177**	0	0	0	0.03333	0	0	0	0	0	0	0	0.03846	0	0	0	0	0	0	0	0
	**178**	0	0	0	0	0	0	0	0	0	0	0	0	0	0	0	0	0.0625	0	0	0
	**179**	0	0.125	0.02632	0.16667	0	0.05556	0	0	0.02083	0	0	0	0.01786	0.03846	0	0	0	0	0.01515	0
	**180**	0	0	0	0	0	0	0	0	0	0	0.08333	0	0	0	0	0	0	0	0	0.10417
	**181**	0.3	0.25	0.31579	0.23333	0	0.11111	0	0	0	0.0625	0.33333	0.07692	0.23214	0.23077	0.08333	0	0.0625	0.05556	0	0.16667
	**182**	0	0	0	0	0	0	0	0	0	0	0	0	0	0	0	0	0	0	0	0
	**183**	0.5	0.375	0.34211	0.2	0	0.38889	0	0	0.08333	0.1875	0	0.19231	0.16071	0.15385	0.08333	0.06818	0.0625	0.16667	0.18182	0.04167
	**184**	0	0	0	0	0	0	0	0	0	0	0	0	0	0	0	0	0	0	0	0
	**185**	0	0.25	0.07895	0.06667	0	0.11111	0	0	0.0625	0.25	0.08333	0.11538	0.07143	0.11538	0.16667	0.04545	0.1875	0.16667	0.04545	0.0625
	**186**	0	0	0	0	0	0	0	0	0	0.0625	0	0.07692	0.08929	0.03846	0	0.06818	0.0625	0.05556	0.04545	0.02083
	**187**	0	0	0	0.03333	0	0.11111	1	0.75	0.04167	0.0625	0.08333	0.15385	0.07143	0	0.08333	0.02273	0.0625	0.16667	0.18182	0
	**188**	0	0	0	0	0	0	0	0	0	0	0	0	0	0	0	0	0	0	0	0
	**189**	0	0	0	0.03333	0	0	0	0	0.02083	0	0	0.03846	0.01786	0	0	0	0	0	0.0303	0
	**191**	0.1	0	0	0	0	0	0	0	0	0	0	0	0	0	0	0.04545	0	0	0.01515	0
	**195**	0	0	0	0	0	0	0	0	0.02083	0	0	0	0	0	0	0	0	0	0	0
	**199**	0	0	0	0	0	0	0	0	0	0	0	0.03846	0	0	0	0	0	0	0	0
